# Neutrophil-specific deletion of the CARD9 gene expression regulator suppresses autoantibody-induced inflammation *in vivo*

**DOI:** 10.1038/ncomms11004

**Published:** 2016-04-01

**Authors:** Tamás Németh, Krisztina Futosi, Cassian Sitaru, Jürgen Ruland, Attila Mócsai

**Affiliations:** 1 Department of Physiology, Semmelweis University School of Medicine, 1094 Budapest, Hungary; 2 MTA-SE ‘Lendület' Inflammation Physiology Research Group of the Hungarian Academy of Sciences and Semmelweis University, 1094 Budapest, Hungary; 3 Department of Dermatology, University Hospital Freiburg and BIOSS Centre for Biological Signalling Studies, 79104 Freiburg, Germany; 4 Department of Clinical Chemistry and Pathobiochemistry, Technical University of Munich, 81675 Munich, Germany

## Abstract

Neutrophils are terminally differentiated cells with limited transcriptional activity. The biological function of their gene expression changes is poorly understood. CARD9 regulates transcription during antifungal immunity but its role in sterile inflammation is unclear. Here we show that neutrophil CARD9 mediates pro-inflammatory chemokine/cytokine but not lipid mediator release during non-infectious inflammation. Genetic deficiency of CARD9 suppresses autoantibody-induced arthritis and dermatitis in mice. Neutrophil-specific deletion of CARD9 is sufficient to induce that phenotype. *Card9*^−/−^ neutrophils show defective immune complex-induced gene expression changes and pro-inflammatory chemokine/cytokine release but normal LTB_4_ production and other short-term responses. *In vivo* deletion of CARD9 reduces tissue levels of pro-inflammatory chemokines and cytokines but not LTB_4_. The CARD9-mediated signalling pathway involves Src-family kinases, Syk, PLCγ2, Bcl10/Malt1 and NFκB. Collectively, CARD9-mediated gene expression changes within neutrophils play important roles during non-infectious inflammation *in vivo* and CARD9 acts as a divergence point between chemokine/cytokine and lipid mediator release.

## Abstract (web summary)

Neutrophils play an important role in antimicrobial host defence but can also contribute to non-infectious inflammatory processes. Here the authors show that adaptor protein CARD9 expressed in neutrophils is involved in the development of sterile auto-antibody-mediated inflammatory reactions.

## Graphical abstract

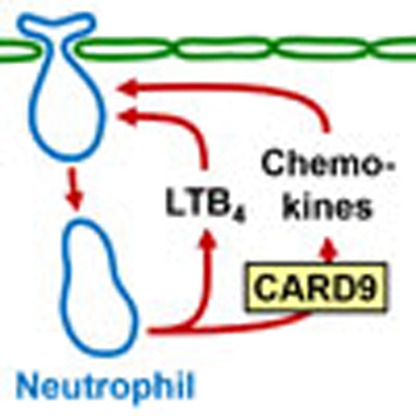

Neutrophils are short-lived, terminally differentiated cells that play critical roles in antimicrobial host defence and also contribute to autoimmune and other non-infectious inflammatory processes^1^,^2^,^3^. Unlike the long-lived macrophages and dendritic cells, neutrophils have limited transcriptional activity but instead show robust short-term effector functions such as phagocytosis, respiratory burst, degranulation or NET release. The dissociation of neutrophil function from gene expression is best exemplified by the fact that anuclear neutrophils that have expelled their DNA through NETosis are still capable of performing various *in vivo* antimicrobial functions^4^. Based on their short lifespan, limited transcriptional activity and robust short-term effector functions, neutrophils are generally believed to be simple effector cells of the immune and inflammatory reaction.

However, neutrophils have also been shown to be able to upregulate pro-inflammatory gene expression and to release various chemokines and cytokines^5^,^6^. Those non-conventional functional responses may indicate a more general role of neutrophils in the orchestration of the immune/inflammatory response^1^,^3^,^6^. Unfortunately, it is still unclear whether inflammation-related gene expression changes in neutrophils (and the resulting chemokine/cytokine production) are just evolutionary remnants from the macrophage-related origin of these cells, or they play an important functional role during the *in vivo* inflammation process. This uncertainty is primarily due to the fact that none of the currently available *in vivo* models allow suppression of gene expression changes in such a manner that it is both selective for neutrophils and it also retains other functional responses of neutrophils intact.

Caspase recruitment domain-containing protein 9 (CARD9) is an intracellular adapter protein primarily expressed in myeloid-lineage cells and couples C-type lectin receptors to NFκB-mediated gene expression^7^. CARD9 plays a critical role in host defence against fungal pathogens in both mice^8^,^9^,^10^ and humans^11^,^12^, and it is also involved in immunity against other microbial infections^7^,^13^. In addition to its antimicrobial function, human genetic studies have also linked CARD9 to highly prevalent human diseases of non-infectious origin such as inflammatory bowel disease^14^,^15^,^16^,^17^, ankylosing spondylitis^18^,^19^, rheumatoid arthritis^20^ or IgA nephropathy^21^. However, it is still unclear whether CARD9 indeed participates in non-infectious inflammation and if so, what are the cellular and molecular pathways involved. In addition, though the analysis of CARD9 function has so far focused on dendritic cells and macrophages, CARD9 is also present in neutrophils^12^,^22^ and the ImmGen database^23^ indicates that neutrophils express the highest level of CARD9 within the immune system. Unfortunately, the role of CARD9 in neutrophils is still very poorly understood.

Autoantibody-induced sterile inflammation is an important component of autoimmune disease pathogenesis. Its experimental models^24^,^25^,^26^ mimic important aspects of human rheumatoid arthritis, bullous pemphigoid and epidermolysis bullosa acquisita. Autoantibody-induced inflammation is mediated by sequential activation of lipid (LTB_4_), cytokine and chemokine cascades^27^. Neutrophils are critically involved in autoantibody-induced sterile inflammation^2^,^28^ and we have previously shown that autoantibody-induced inflammation is mediated by signalling through Src-family kinases, Syk and PLCγ2 (refs 29, 30, 31, 32). However, it is at present unclear how signalling downstream of those receptor-proximal molecules triggers lipid, chemokine and cytokine release.

The lack of knowledge on the contribution of neutrophil gene expression to *in vivo* inflammation, on the role of CARD9 in non-infectious inflammation and neutrophil function and on how receptor-proximal signalling molecules are coupled to inflammatory mediator release, prompted us to investigate the role of CARD9 in autoantibody-mediated *in vivo* inflammation models. Our results indicate that CARD9 mediates autoantibody-induced inflammation by acting as a divergence point downstream of receptor-proximal signalling molecules triggering chemokine and cytokine but not lipid mediator (LTB_4_) release. Importantly, lineage-specific studies revealed that those functions are primarily linked to CARD9 expression within neutrophils, indicating a critical contribution of neutrophil gene expression and chemokine/cytokine release to the overall *in vivo* inflammatory reaction.

## Results

### The role of CARD9 in autoantibody-induced arthritis

**Figure 1:**
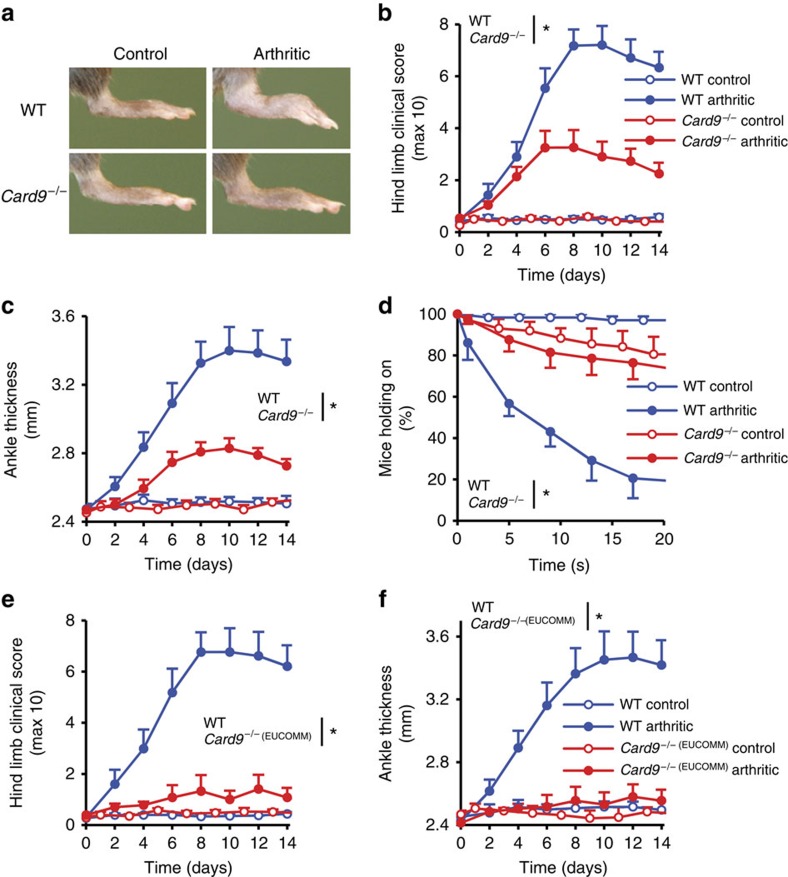
CARD9 is important for the development of autoantibody-induced arthritis. Intact wild type (WT), *Card9*^−/−^ (**a**–**d**) or *Card9*^−/− (EUCOMM)^ (**e**,**f**) mice were injected with B × N (control) or K/B × N (arthritic) serum i.p. on day 0. Arthritis development was followed by photographing on day 7 (**a**), clinical scoring of the hind limbs (**b**,**e**), ankle-thickness measurement (**c**,**f**) and an articular function test (hanging on a wire grid; (**d**)). Images are representative of, and quantitative data show mean and s.e.m. from 8 to 9 control and 12 to 13 arthritic serum-treated individual mice per group from four independent experiments (**a**–**d**) or 5 to 6 control and 8 to 9 arthritic serum-treated mice per group from three independent experiments (**e,f**). (**d**) Results from functional test performed 12 times on each mouse between days 7–10. *P<0.05 (two-way ANOVA); see the text for actual *P* values.

To test the role of CARD9 in non-infectious inflammation, we tested the effect of CARD9 deficiency on autoantibody-induced arthritis development in the K/B × N serum-transfer model^30^. We have used two independent CARD9-deficient mouse strains (Supplementary Fig. 1a) that were homozygous either for the conventional *Card9*^tm1Jrld^ knockout mutation (referred to as *Card9*^−/−^ mice) or for the so-called knockout-first^33^ *Card9*^tm1a(EUCOMM)Hmgu^ mutation (referred to as *Card9*^−/− (EUCOMM)^ mice). CARD9 was absent from bone marrow (Supplementary Fig. 1b) and spleen (Supplementary Fig. 1c) cell lysates of both mutant strains. As indicated in Fig. 1a, *Card9*^−/−^ mice showed substantially reduced signs of arthritis development. Quantification of the visible clinical signs (Fig. 1b; *P*=0.0028; two-way analysis of variance (ANOVA)), ankle thickening (Fig. 1c; *P*=0.0047; two-way ANOVA) and the ability of the mice to hold on to the bottom of a wire grid (Fig. 1d; *P*=2 × 10^−4^; two-way ANOVA) all confirmed highly significant protection from disease development. Similarly, *Card9*^−/− (EUCOMM)^ mice were also strongly protected from visible signs of arthritis (Fig. 1e; *P*=6.6 × 10^−4^; two-way ANOVA) and ankle thickening (Fig. 1f; *P*=0.0038; two-way ANOVA). Therefore, CARD9 plays a major role in the development of autoantibody-induced arthritis.

We have also generated bone marrow chimeras by transplanting CARD9-deficient bone marrow cells to lethally irradiated wild-type recipients. Similar to intact mice, *Card9*^−/−^ bone marrow chimeras also showed partial protection from arthritis development in the K/B × N serum-transfer model (Supplementary Figs 2a–d; *P* values between 9.1 × 10^−11^ and 1.6 × 10^−4^; two-way ANOVA), indicating a role for CARD9 in a radiosensitive hematopoietic compartment.

### The role of CARD9 in autoantibody-induced skin blistering

**Figure 2:**
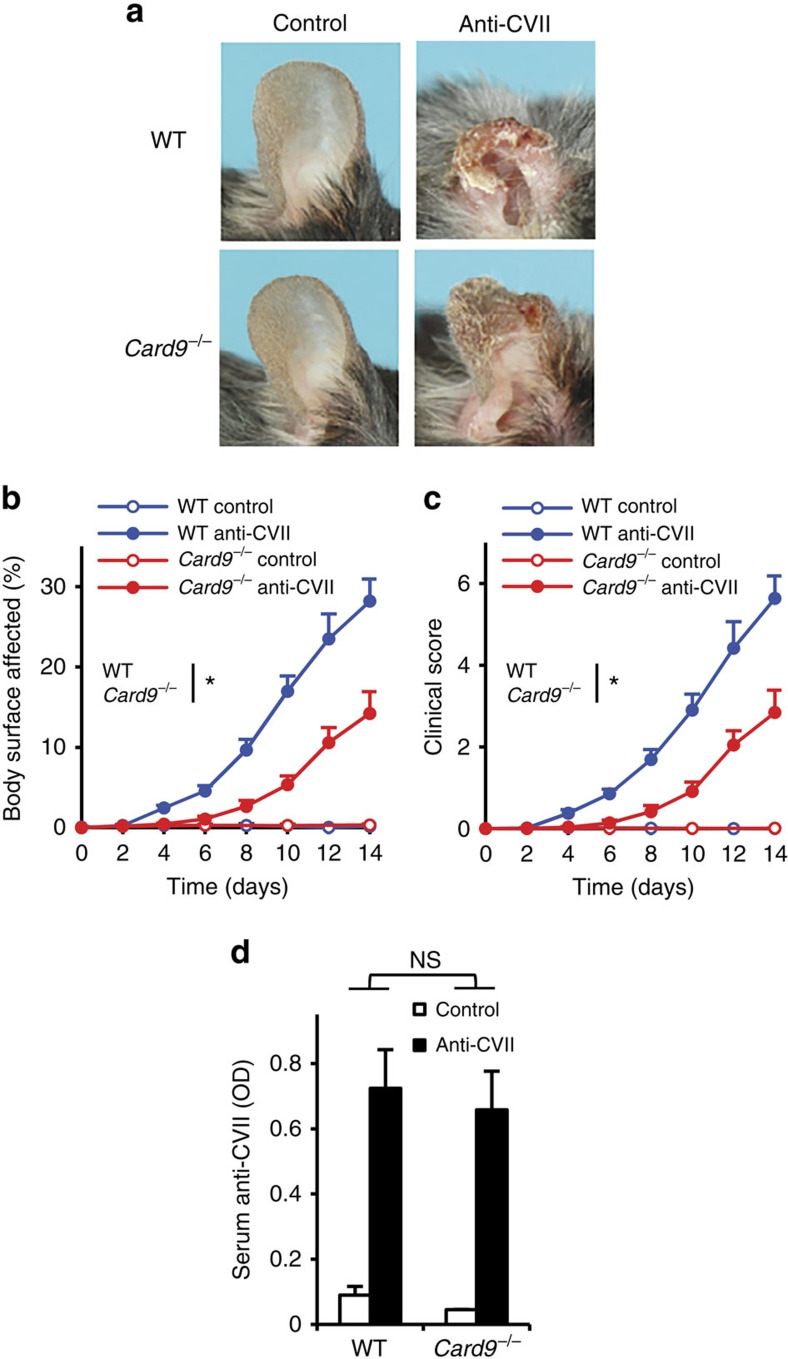
CARD9 in autoantibody-induced skin blistering disease. Blistering skin disease was triggered in wild type (WT) and *Card9*^−/−^ intact mice or bone marrow chimeras by systemic injection of collagen VII-specific (anti-CVII) antibodies. Skin disease was followed by photographing on day 14 (**a**) and clinical assessment of the total body surface affected (**b**) and the overall disease severity (**c**). The serum titre of anti-CVII antibodies was tested on day 6 by ELISA (**d**). Representative images (**a**) or mean and s.e.m. (**b**–**c**) from 7 to 9 control (2 intact and 5–7 bone marrow chimeric) and 13–14 anti-CVII-treated (2 intact and 11–12 bone marrow chimeric) mice per genotype from four independent experiments are shown. No difference between intact and chimeric mice of the same hematopoietic genotype was observed (not shown). (**d**) Mean and s.e.m. from 4 control and 12–13 anti-CVII-treated mice from five independent experiments are shown. *P<0.05; NS, statistically not significant (two-way ANOVA); see the text for actual *P* values.

We have also tested the effect of CARD9 deficiency on dermatitis triggered by systemic administration of antibodies against collagen VII (a mouse model of the human blistering skin disease epidermolysis bullosa acquisita). *Card9*^−/−^ mice were substantially, though not completely protected from the visible skin disease (Fig. 2a), which was also confirmed by quantitative analysis of the body surface area affected (Fig. 2b; *P*=0.017; two-way ANOVA) and the overall disease severity score (Fig. 2c; *P*=0.022; two-way ANOVA). This was not due to the reduced circulating autoantibody concentration since serum levels of collagen VII-specific IgG were similar in wild-type and *Card9*^−/−^ mice (Fig. 2d; *P*=0.95; two-way ANOVA).

### Role of CARD9 in leukocyte recruitment and migration

**Figure 3:**
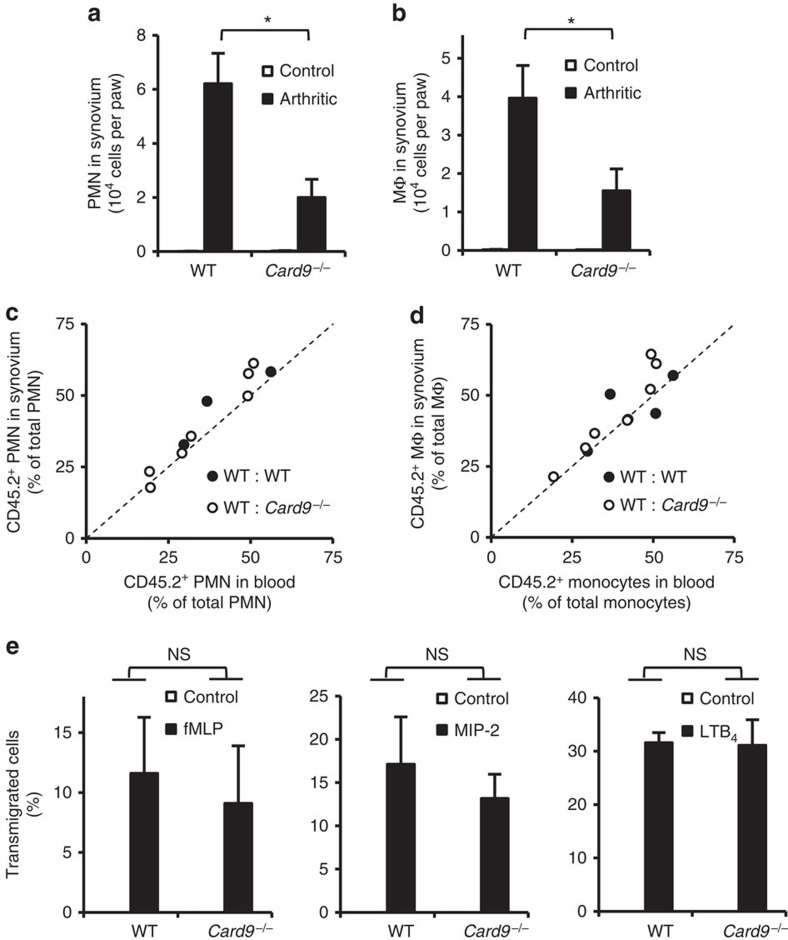
CARD9 plays no direct role in myeloid cell migration to the site of inflammation. Wild type (WT) and *Card9*^−/−^ mice were subjected to K/B × N serum-transfer arthritis (**a**–**d**), the ankle area or the front paw was flushed on day 4 and the number of neutrophils (**a**) or monocytes/macrophages (**b**) was determined by flow cytometry. Mixed bone marrow chimeras with CD45.1-expressing wild type (WT) and CD45.2-expressing WT or *Card9*^−/−^ hematopoietic cells were subjected to K/B × N serum-transfer arthritis and *in vivo* accumulation of neutrophils (**c**) and monocytes/macrophages (**d**) was determined by flushing the synovial area on day 4, followed by flow cytometric analysis of the ratio of CD45.1 and CD45.2-expressing cells in peripheral blood and the synovial infiltrate. In (**c,d**), each dot represents an individual mouse. (**e**) *In vitro* migration of WT and *Card9*^−/−^ bone marrow neutrophils towards the indicated chemoattractants in a fibrinogen-coated Transwell system. Graphs represent mean and s.e.m. from 5–14 (**a**) or 6–16 (**b**) mice per group from five independent experiments. (**e**) Data represent mean and s.e.m. of three to five independent experiments. *P<0.05; NS, statistically not significant (Student's *t*-test (**a**,**b**), two-way ANOVA (**e**)); see the text for actual *P* values.

Flow cytometric analysis of myeloid cells in the synovial infiltrate (Fig. 3a,b) revealed reduced accumulation of neutrophils (*P*=0.0041; Student's *t*-test) and macrophages (*P*=0.026; Student's *t*-test) at the site of inflammation in *Card9*^−/−^ mice subjected to K/B × N serum-transfer arthritis. To reveal whether that reduction was due to a cell-autonomous migration defect, we generated mixed bone marrow chimeras carrying CD45.1-expressing wild type, along with CD45.2-expressing wild type or *Card9*^−/−^ hematopoietic tissues (WT: WT and WT: *Card9*^−/−^ chimeras, respectively). Those chimeras were subjected to K/B × N serum-transfer arthritis and the ability of myeloid cells from the different genotypes to infiltrate the inflamed synovium was tested by comparing the ratio of CD45.1-expressing and CD45.2-expressing cells in the peripheral blood and synovial infiltrate. As shown in Fig. 3c and Supplementary Fig. 3a, no significant difference between the percentage of CD45.2-positive neutrophils in the blood and synovium was observed in any of the mixed bone marrow chimeras irrespective of the genotype (wild type or *Card9*^−/−^) of the CD45.2-positive population (*P*=0.80 for normalized data shown in Supplementary Fig. 3a; Student's *t*-test). Similarly, the percentage of CD45.2-positive monocytes in the blood was always similar to that of CD45.2-positive macrophages in the synovium, irrespective of the genotype of the CD45.2-positive cells (Fig. 3d and Supplementary Fig. 3b; *P*=0.56; Student's *t*-test). Those results indicated that *Card9*^−/−^ myeloid cells did not have an intrinsic migration defect *in vivo*.

We also tested the intrinsic migratory capacity of neutrophils in *in vitro* transwell assays. As shown in Fig. 3e, the *Card9*^−/−^ mutation did not affect the migration of neutrophils towards fMLP (*P*=0.71; two-way ANOVA), MIP-2 (CXCL2; *P*=0.53; two-way ANOVA) or LTB_4_ (*P*=0.92; two-way ANOVA) as chemoattractants either.

Taken together, *Card9*^−/−^ myeloid cells show partially reduced accumulation at the site of inflammation despite no cell-autonomous migration defect.

### Decreased chemokine and cytokine but not LTB_4_ levels *in vivo*

**Figure 4:**
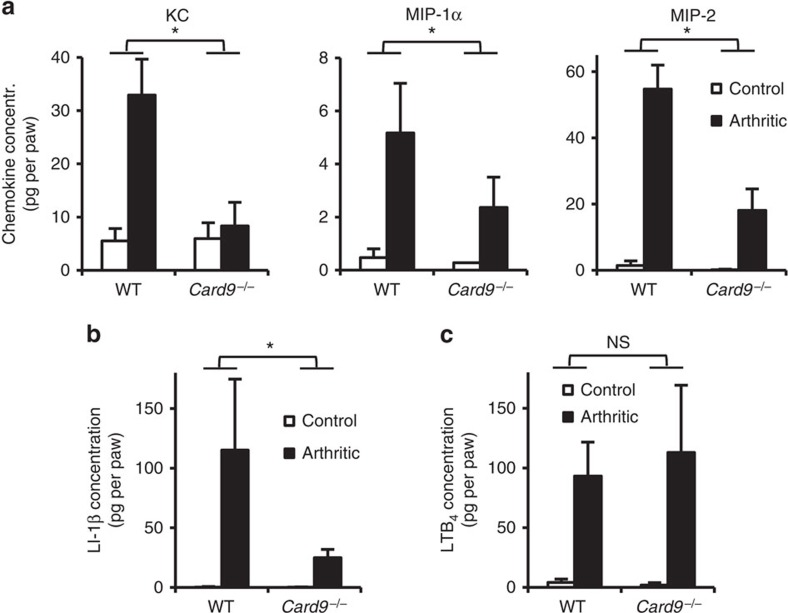
CARD9 is required for the development of the inflammatory environment. (**a**–**c**) Wild type (WT) or *Card9*^−/−^ mice were subjected to K/B × N serum-transfer arthritis as described above and the synovial area was flushed on day 4. The cell-free supernatants of the synovial infiltrates were probed using commercial ELISA assays for the indicated chemokines (**a**), cytokine (**b**) and the lipid mediator LTB_4_ (**c**). Graphs represent mean and s.e.m. from 4–9 (**a**), 3–5 (**b**) or 3–4 (**c**) mice per group from three independent experiments. *P<0.05; NS, statistically not significant (two-way ANOVA); see the text for actual *P* values.

To resolve the apparent contradiction between partially reduced accumulation but normal intrinsic migratory capacity of *Card9*^−/−^ myeloid cells, we hypothesized that CARD9 was required for the generation of an appropriate inflammatory (chemoattractant) tissue microenvironment. Therefore, we tested the presence of various pro-inflammatory mediators in the synovial infiltrate. As shown in Fig. 4a,b, the *Card9*^−/−^ mutation led to a strong reduction of the levels of various chemokines acting on myeloid cells, including KC (CXCL1; *P*=0.011; two-way ANOVA), MIP-1α (CCL3; *P*=0.0013; two-way ANOVA) and MIP-2 (CXCL2; *P*=0.027; two-way ANOVA), as well as the IL-1β cytokine (*P*=2.5 × 10^−5^; two-way ANOVA). Interestingly, however, the *Card9*^−/−^ mutation did not affect the level of the lipid chemoattractant LTB_4_ at the site of inflammation (Fig. 4c; *P*=0.77; two-way ANOVA). Those results likely explain the substantially but not completely reduced accumulation of myeloid cells in the inflamed synovium.

### Neutrophil CARD9 is required for *in vivo* inflammation

The importance of neutrophils in autoantibody-induced inflammation^28^,^34^ and the apparent role of CARD9 in a radiosensitive hematopoietic compartment (Supplementary Fig. 2) raised the possibility that CARD9 expressed in neutrophils was required for the autoantibody-induced arthritis and dermatitis response. The availability of the *Card9*^tm1a(EUCOMM)Hmgu^ allele that allows further manipulation towards lineage-specific deletion provided a unique opportunity to test the functional relevance of CARD9 specifically within the neutrophil lineage.

**Figure 5:**
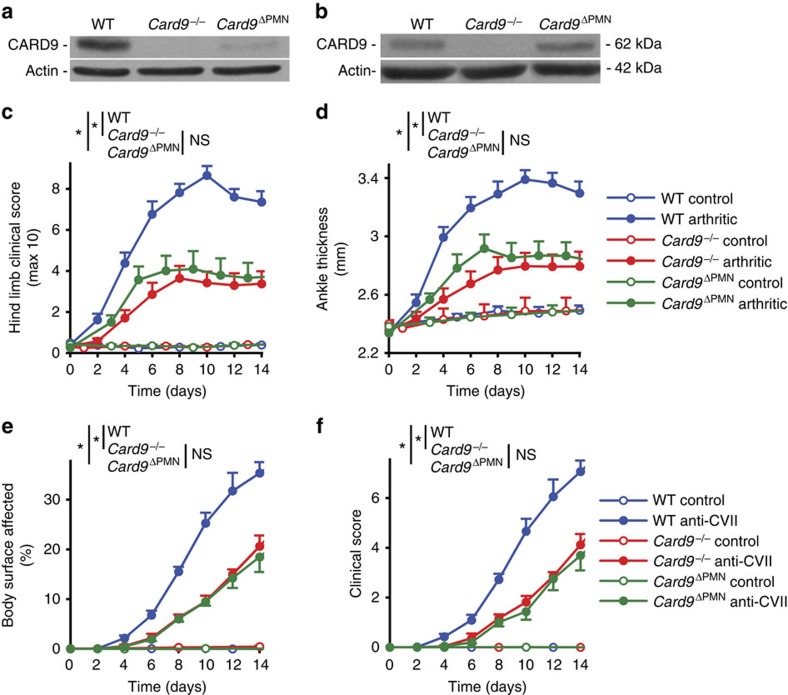
Neutrophil-specific CARD9 deletion attenuates autoantibody-induced inflammation. (**a**,**b**) CARD9 expression was detected by western blot in cell-sorted bone marrow-derived neutrophils (**a**) or cultured macrophages (**b**) of wild type, *Card9*^−/−^ or *Card9*^ΔPMN^ animals. (**c**,**d**) Wild type (WT), *Card9*^−/−^ or *Card9*^ΔPMN^ bone marrow chimeras were injected with B × N (control) or K/B × N (arthritic) serum i.p. on day 0. Arthritis development was followed by clinical scoring of the hind limbs (**c**) and ankle-thickness measurement (**d**). Blistering skin disease was triggered in wild type (WT), *Card9*^−/−^ or *Card9*^ΔPMN^ intact mice or bone marrow chimeras by systemic injection of collagen VII-specific (anti-CVII) antibodies. Skin disease was followed by clinical assessment of the total body surface affected (**e**) and the overall disease severity (**f**). The blots are representative of three to four independent experiments. Quantitative data show mean and s.e.m. from 7 control and 8 to 9 arthritic serum-treated individual mice per group from three independent experiments (**c**,**d**) or 3 to 4 control and 4 anti-CVII-treated mice per group from two independent experiments (**e**,**f**). *P<0.05 (two-way ANOVA); NS, not significant; see the text for actual *P* values.

Using FRT-mediated manipulation of the *Card9*^tm1a(EUCOMM)Hmgu^ knockout-first allele (Supplementary Fig. 4), we have generated mice carrying the *Card9*^tm1c(EUCOMM)Hmgu^ (referred to as *Card9*^flox^) floxed allele and bred those mice to MRP8-Cre^35^ transgene-expressing mice to generate MRP8-Cre^+^*Card9*^flox/flox^ (referred to as *Card9*^ΔPMN^) mice with lineage-specific deletion of CARD9 specifically in the neutrophil compartment (Supplementary Fig. 4). As shown in Fig. 5a, FACS-sorted *Card9*^−/−^ neutrophils had no detectable CARD9 expression, whereas the level of CARD9 was strongly reduced (though not completely absent) in similarly sorted *Card9*^ΔPMN^ neutrophils. However, in agreement with prior studies showing high selectivity of the MRP8-Cre transgene^35^,^36^, the *Card9*^ΔPMN^ mutation did not affect CARD9 expression in bone marrow-derived macrophages (Fig. 5b). Those results confirmed the effective and specific deletion of CARD9 from *Card9*^ΔPMN^ neutrophils.

We next tested the effect of the *Card9*^ΔPMN^ mutation on K/B × N serum-transfer arthritis. As shown in Fig. 5c, both the *Card9*^−/−^ (*P*=1.4 × 10^−6^; two-way ANOVA) and the *Card9*^ΔPMN^ (*P*=4 × 10^−4^; two-way ANOVA) mutations caused a substantial but incomplete reduction of the clinical arthritis score. Similarly, the ankle-thickening response (Fig. 5d) was reduced in both *Card9*^−/−^ (*P*=4.3 × 10^−4^; two-way ANOVA) and *Card9*^ΔPMN^ (*P*=0.0069; two-way ANOVA) mutants. Importantly, there was practically no difference between the effect of neutrophil-specific and complete CARD9 deficiency in either assay readouts tested (*P* values between 0.47 and 0.56; two-way ANOVA).

We have also subjected the same mutants to the autoantibody-induced skin blistering disease model. As shown in Fig. 5e, both the *Card9*^−/−^ (*P*=4.6 × 10^−6^; two-way ANOVA) and the *Card9*^ΔPMN^ (*P*=1.7 × 10^−5^; two-way ANOVA) mutants were significantly but not completely protected from disease development defined as the body area affected. Similarly, the clinical score (Fig. 5f) was reduced in both *Card9*^−/−^ (*P*=1.6 × 10^−4^; two-way ANOVA) and *Card9*^ΔPMN^ (*P*=7.5 × 10^−5^; two-way ANOVA) mutants. Again, the two mutant strains showed practically identical phenotypes in both assay readouts tested (*P* values between 0.17 and 0.69; two-way ANOVA).

Taken together, the defective arthritis and dermatitis development in *Card9*^−/−^ mice is mostly due to the role of CARD9 within the neutrophil compartment.

### Reduced neutrophil chemokine/cytokine but not LTB_4_ release

Given the *in vivo* role of CARD9 within neutrophils, we next tested various *in vitro* functions of *Card9*^−/−^ neutrophils. Those experiments focused on immune complex-induced activation as an *in vitro* model of autoantibody-induced *in vivo* activation.

Although *Card9*^−/−^ neutrophils lacked the CARD9 protein (Supplementary Fig. 5a), they expressed normal levels of the neutrophil maturation marker Ly6G (Supplementary Fig. 5b), and showed normal expression of cell surface FcγRII/III and FcγRIV (Supplementary Fig. 5c).

**Figure 6:**
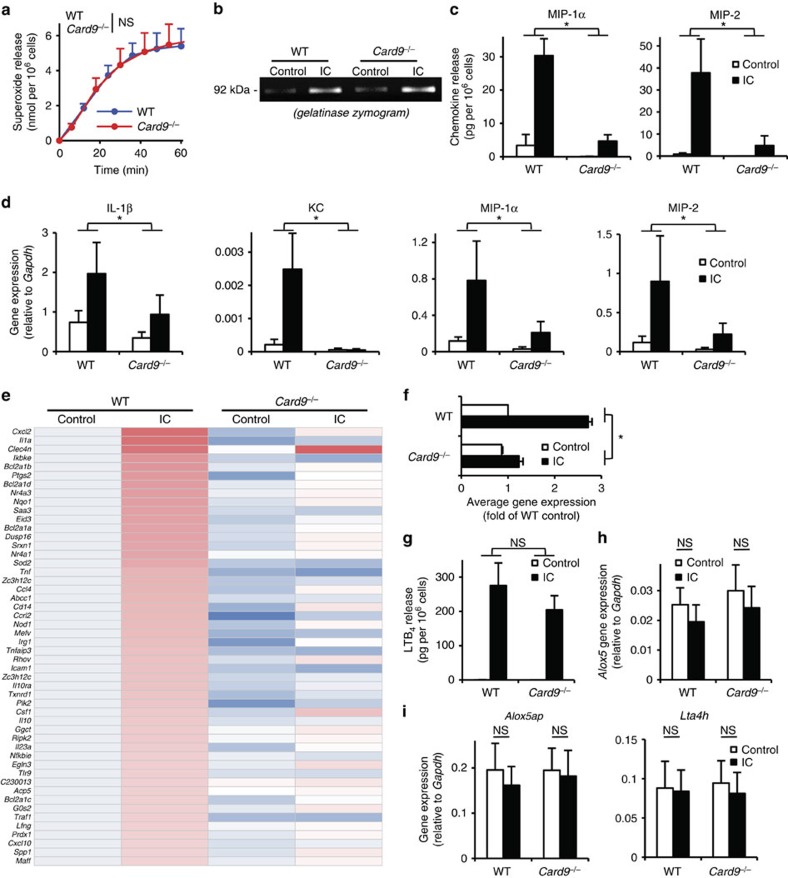
CARD9 is required for neutrophil gene expression changes and chemokine/cytokine release. Wild type (WT) and *Card9*^*−/−*^neutrophils were placed on immobilized immune complex (IC) surfaces. Superoxide release (**a**) was followed by a spectrophotometric assay, while gelatinase degranulation was determined by gelatinase zymography (**b**). Cytokine, chemokine and lipid mediator levels in cell-free supernatants were determined after an incubation for 6 h (cytokines) or 1 h (LTB_4_) using ELISA assays (**c**,**g**). Gene expression changes were followed by quantitative PCR (**d**,**h**,**i**) or by Affymetrix Microarrays (**e**,**f**). (**f**) The average changes of the expression of the 50 most highly upregulated genes shown in (**e**). Kinetic curves in **a** show mean and s.e.m. of six independent experiments. Control data points were subtracted. (**b**) Representative of three independent experiments. Graphs (**c**,**d**,**f**–**i**) show mean and s.e.m. from three to five independent experiments, while the heat map in **e** represents the colour-coded mean of three independent experiments. *P<0.05; NS, statistically not significant (two-way ANOVA except for **h** and **i** where one-way ANOVA was used); see the text for actual *P* values.

CARD9 deficiency did not affect immune complex-induced short-term functional responses of neutrophils such as superoxide release (Fig. 6a; *P*=0.79; two-way ANOVA) or the exocytosis of gelatinase-containing granules (Fig. 6b). However, CARD9 deficiency markedly reduced the immune complex-induced release of MIP-1α (*P*=0.0079; two-way ANOVA) and MIP-2 (*P*=1.2 × 10^−7^; two-way ANOVA) chemokines from neutrophils (Fig. 6c). As shown in Fig. 6d, *Card9*^−/−^ neutrophils also showed strong reduction of the immune complex-induced expression of the genes encoding for IL-1β (*P*=0.018; two-way ANOVA), KC (*P*=6.0 × 10^−16^; two-way ANOVA), MIP-1α (*P*=9.6 × 10^−7^; two-way ANOVA) and MIP-2 (*P*=6.2 × 10^−8^; two-way ANOVA).

We have also performed complementary DNA (cDNA) microarray analysis to test whether the reduced upregulation of chemokine and cytokine genes reflected a generalized defect of immune complex-induced gene expression (Fig. 6e,f; Supplementary Table 1). As shown in the heat map of the 50 most highly upregulated genes (Fig. 6e), CARD9 deficiency led to an overall decrease of immune complex-induced upregulation of neutrophil gene expression, including expression of various genes expressing pro-inflammatory mediators. The average changes of the expression of the genes shown in Fig. 6e has also been summarized in Fig. 6f, which indicated a 78% reduction of immune complex-induced gene expression in *Card9*^−/−^ cells (*P*<10^-17^; two-way ANOVA).

In contrast to the various chemokines and cytokines, the immune complex-induced release of LTB_4_ from neutrophils was not affected by the *Card9*^−/−^ mutation (Fig. 6g; *P*=0.39; two-way ANOVA). In addition, the expression of *Alox5* that encodes for 5-lipoxigenase, the rate-limiting enzyme of LTB_4_ synthesis, is not triggered by immune complex stimulation in wild-type neutrophils (*P*=0.56; one-way ANOVA) and it is not affected by the *Card9*^−/−^ mutation either (Fig. 6h). The expression of *Alox5ap* and *Lta4h*, the other two components of LTB_4_ synthesis, was also not altered by immune complex stimulation (*P*=0.49 and 0.64, respectively; one-way ANOVA) or CARD9 deficiency (Fig. 6i).

Taken together, CARD9 is required for immune complex-induced gene expression changes and release of various chemokines and cytokines but not LTB_4_ from neutrophils. Together with the defective inflammatory response in neutrophil-specific CARD9 deletion mutants (Fig. 5), these results also provide strong evidence for the *in vivo* role of gene expression changes within the neutrophil compartment.

### Upstream and downstream signalling components

**Figure 7:**
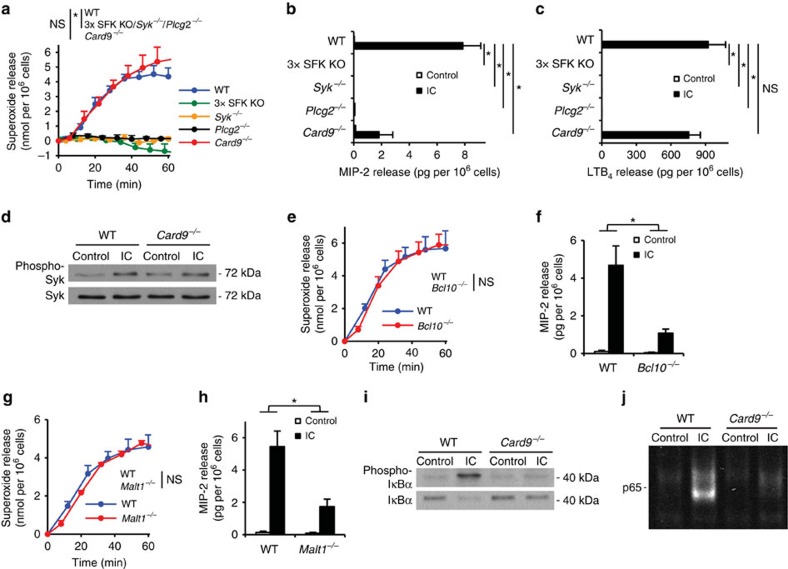
Upstream and downstream components of the CARD9 signalling pathway. (**a**–**c**) Wild type (WT), *Hck*^−/−^*Fgr*^−/−^*Lyn*^−/−^ (3 × SFK KO), Syk^−/−^, *Plcg2*^−/−^ or *Card9*^−/−^ neutrophils were placed on immobilized immune complex (IC) surfaces. Their superoxide release (**a**), MIP-2 levels (**b**) and LTB_4_ (**c**) release were determined. (**d**) WT and *Card9*^−/−^ neutrophils were plated on IC surfaces for 10 min, the phosphorylation and the total amount of Syk in cell lysates was determined by western blot after immunoprecipitation. (**e**–**h**) Bcl10^−/−^ and Malt1^−/−^ neutrophils showed similar functional phenotypes on IC surfaces like *Card9*^−/−^ neutrophils: they had intact superoxide release (**e**,**g**) and defective chemokine production (**f**,**h**). (**i**) WT and *Card9*^−/−^ neutrophils were stimulated for 20 min; the phosphorylation and degradation of IκBα was followed by western blotting from whole cell lysates. (**j)** IC-activated WT and *Card9*^−/−^ neutrophils were lysed after 20 min; the activation and nuclear translocation of NF-κB was determined by infrared dye-labelled NFκB consensus binding site probes. Kinetic curves in **a**,**e** and **g** show mean and s.e.m. of three to four independent experiments. Control data points were subtracted. Graphs in **b**,**c**,**f** and **h** represent data from two to four independent experiments. (**d**,**i,j**) Representative of two to three independent experiments. *P<0.05; NS, statistically not significant (two-way ANOVA); see the text for actual *P* values.

Having established a critical role for CARD9-mediated neutrophil gene expression changes and chemokine/cytokine release, we finally aimed to characterize the signalling components upstream and downstream of CARD9 in neutrophils. We and others have previously shown that myeloid Src-family kinases (Hck, Fgr and Lyn)^32^, Syk^31^,^37^ and PLCγ2 (refs 29, 30) are required for immune complex-induced neutrophil functions and the K/B × N serum-transfer arthritis, supposedly by forming a receptor-proximal signalling cluster. We therefore compared immune complex-induced functional responses of neutrophils lacking those molecules with responses of *Card9*^−/−^ cells. As shown in Fig. 7a, *Hck*^−/−^*Fgr*^−/−^*Lyn*^−/−^ (3 × SFK KO), *Syk*^−/−^ and *Plcg2*^−/−^ neutrophils showed complete defect in immune complex-induced superoxide production (*P* values between 7.9 × 10^−5^ and 1.6 × 10^−4^; two-way ANOVA), whereas *Card9*^−/−^ cell showed a normal response (*P*=0.73; two-way ANOVA). Similarly, as shown in Fig. 7b,c, while *Hck*^−/−^*Fgr*^−/−^*Lyn*^−/−^, *Syk*^−/−^ and *Plcg2*^−/−^ neutrophils showed complete defect in immune complex-induced MIP-2 and LTB_4_ release (*P* values between 2.9 × 10^−4^ and 9.4 × 10^−3^; two-way ANOVA), *Card9*^−/−^ neutrophils displayed a strong but incomplete reduction of MIP-2 release (*P*=0.027; two-way ANOVA) and no significant reduction of LTB_4_ release (*P*=0.38; two-way ANOVA). The *Card9*^−/−^ neutrophil phenotype was not due to defective receptor-proximal signalling since *Card9*^−/−^ neutrophils showed normal Syk phosphorylation on activation by immobilized immune complexes (Fig. 7d). Those results suggest that CARD9 functions downstream of the receptor-proximal Src-family/Syk/PLCγ2 complex during immune complex-induced neutrophil activation.

During fungal infection, CARD9 coordinates anti-fungal host defence in cooperation with the downstream adapter Bcl10 (ref. 7). Therefore, we tested neutrophil functions and arthritis development in the absence of Bcl10. *Bcl10*^−/−^ neutrophils lacked the Bcl10 protein (Supplementary Fig. 6a) but expressed normal levels of Ly6G (Supplementary Fig. 6b) and Fcγ-receptors (Supplementary Fig. 6c). On immune complex challenge, *Bcl10*^−/−^ neutrophils showed normal superoxide release (Fig. 7e; *P*=0.76; two-way ANOVA) but defective release of the MIP-2 chemokine (Fig. 7f; *P*=0.0057; two-way ANOVA). As shown in Supplementary Fig. 7, the *Bcl10*^−/−^ mutation also caused partial protection from the clinical signs (*P*=0.065; two-way ANOVA) and ankle-thickening response (*P*=0.015; two-way ANOVA) during K/B × N serum-transfer arthritis. The CARD9-Bcl10 complex is thought to signal through the Malt1 paracaspase during antifungal immunity^7^. As shown in Fig. 7g,h, *Malt1*^−/−^ neutrophils produced normal levels of superoxide on immune complex activation (*P*=0.87; two-way ANOVA) but their MIP-2 release was substantially reduced under identical conditions (*P*=0.0092; two-way ANOVA). The similarity between the *Card9*^−/−^, *Bcl10*^−/−^ and *Malt1*^−/−^ phenotypes suggests that Bcl10 and Malt1 participates in signalling downstream of CARD9 during immune complex-induced neutrophil activation.

We have also explored how CARD9 is coupled to upregulation of pro-inflammatory gene expression. CARD9 deficiency strongly reduced immune complex-induced phosphorylation and degradation of IκBα (Fig. 7i) and the translocation of NFκB p65 to the nucleus (Fig. 7j), indicating that CARD9 triggers upregulation of inflammatory gene expression through activation of the canonical NFκB pathway.

Taken together, the CARD9 signalling pathway responsible for chemokine/cytokine production by neutrophils during inflammation *in vivo* is reminiscent of the signalling pathway utilized by C-type lectin receptors and consists of a receptor-proximal Src-family/Syk/PLCγ2 module triggering NFκB activation through the CARD9/Bcl10/Malt1 complex.

## Discussion

Our results indicate that the CARD9 protein expressed within the neutrophil compartment plays an important role in chemokine and cytokine but not LTB_4_ release during autoantibody-induced sterile inflammation. To our knowledge, these are the first experiments showing that neutrophil-specific abrogation of agonist-induced gene expression changes (without affecting other neutrophil functions) inhibits *in vivo* inflammatory reactions and therefore provide the most direct evidence so far for an important *in vivo* role of inflammation-induced gene expression changes within the neutrophil lineage.

Although neutrophils have long been known to be critical effector cells of antimicrobial host defence, more recent studies indicated that they are also capable of releasing pro-inflammatory mediators^5^,^6^. Unfortunately, direct evidence for the role of neutrophil gene expression or neutrophil-derived chemokines/cytokines has been missing. A number of prior studies reported a critical role for neutrophils in *in vivo* inflammatory reactions^2^,^28^ and a recent study has shown that neutrophil-specific deletion of Syk blocked arthritis development in the K/B × N serum-transfer model^37^. Unfortunately, since those approaches not only blocked neutrophil-derived gene expression but also other functional responses of those cells, the specific effect of gene expression changes could not be assessed. Other attempts utilized complementation approaches using mixed bone marrow chimeras or isolated bone marrow neutrophils^27^,^38^,^39^. Unfortunately, all those approaches had major limitations related to the limited specificity of the *Gfi1*^−/−^ strain used or the limited purity and altered activation status of *ex vivo* isolated bone marrow-derived neutrophils. Our results with the *Card9*^ΔPMN^ mutants (Fig. 5) provide the first direct evidence using lineage-specific deletion that the products of neutrophil gene expression coordinate the *in vivo* inflammatory response without affecting conventional neutrophil functions such as reactive oxygen species (ROS) release. Our overall study also suggests that neutrophil-derived LTB_4_ is capable of driving a clinically evident, though substantially reduced level of neutrophil-mediated inflammation even in the absence of neutrophil-derived chemokines and cytokines. Those results together provide substantial novel insight into the *in vivo* role of neutrophil gene expression and neutrophil-derived mediators during the inflammatory response.

The role of CARD9 has so far mainly been tested in functional responses of dendritic cells and macrophages, myeloid cell types primarily implicated in the organization of an adaptive immune response. Though we have observed that *Card9*^−/−^ macrophages also show defective chemokine but not ROS release (Supplementary Fig. 8), our lineage-specific studies indicate that, at least in our experimental system, the effect of CARD9 deficiency is primarily due to the CARD9 expression in neutrophils. This is further supported by the presence of normal levels of CARD9 in *Card9*^ΔPMN^ macrophages.

A crucial question related to the interpretation of our lineage-specific deletion studies is the specificity of the MRP8-Cre transgene. The MRP8 (S100A8) protein is also expressed in other myeloid cells such as macrophages, and therefore Cre-mediated deletion of target genes could have been predicted also in macrophages. However, the fact that CARD9 is expressed normally in *Card9*^ΔPMN^ bone marrow-derived (Fig. 5b) macrophages indicates that this is not the case. Indeed, the original description of the MRP8-Cre transgene^35^ indicated high specificity towards neutrophils and detailed recent studies have also shown negligible deletion of floxed genomic sequences in MRP8-Cre transgene-positive macrophages or dendritic cells^36^,^37^. This high specificity may be due to the fact that MRP8 is likely the most abundant cytoplasmic protein in neutrophils and the ImmGen database^23^ indicates that it is expressed at 20–100-fold higher levels in neutrophils than in any other myeloid lineage cell types. Those results together provide convincing evidence that the *in vivo* phenotypes of the *Card9*^ΔPMN^ mutants are due to specific deletion of CARD9 from the neutrophil lineage.

Although the role of CARD9 in antifungal and other antimicrobial immunity is well established^7^, its role in non-infectious inflammation is poorly understood. The protection of *Card9*^−/−^ mice from autoantibody-induced inflammatory reactions and the defective responses of *Card9*^−/−^ cells to immune complex stimulation indicate a critical role for CARD9 also in non-infectious inflammatory processes. This issue is particularly important given the association of CARD9 polymorphisms with rheumatoid arthritis, inflammatory bowel disease, ankylosing spondylitis or IgA nephropathy^14^,^15^,^16^,^17^,^18^,^19^,^20^,^21^, major human inflammatory diseases of non-infectious origin. IgA nephropathy, the most frequent form of primary glomerulonephritis in humans, is particularly interesting since it is caused by immune complex deposition at the glomerular basement membrane^40^. As neutrophils are the primary cell types responding to systemic IgA through the human-specific FcαRI (CD89) molecule^41^, it is tempting to speculate that, similar to our studies in mice, the potential role of CARD9 in IgA nephropathy is due to its role in immune complex-induced neutrophil activation.

Autoantibody-induced inflammation has been shown to be mediated by a lipid-cytokine-chemokine cascade^27^, which also triggers autoamplification of leucocyte recruitment^42^. We and others have previously shown that a receptor-proximal Src-family/Syk/PLCγ2 signalling module is critical for autoantibody-induced arthritis and dermatitis^29^,^30^,^31^,^32^,^37^,^43^. However, it has been unclear how that signalling module is coupled to chemokine, cytokine and lipid mediator release. Our results indicate that CARD9 acts as a divergence point downstream of the receptor-proximal Src-family/Syk/PLCγ2 module in neutrophils and leads to Bcl10/Malt1- and NFκB-dependent chemokine and cytokine release without playing a role in lipid mediator (LTB_4_) production (Supplementary Fig. 9). Those results reveal intricate details of myeloid cell signalling during autoantibody-induced inflammation. The normal LTB_4_ release also likely explains why *Card9*^−/−^ animals are only partially protected from arthritis and dermatitis development.

Though it is not entirely clear how the CARD9–Bcl10–Malt1 complex activates the NFκB pathway, the mechanism of activation and nuclear translocation of NFκB by the CARD9-related CARMA-like proteins has been investigated in other immune cells. Assembly of CARMA proteins with Bcl10 and Malt1 leads to the ubiquitination of the regulatory IκBα kinase subunit NEMO and the phosphorylation and degradation of IκBα(ref 44). CARMA-like proteins supposedly exert their effect through binding to TRAF6 and recruiting TAK1 to the CARMA-Bcl10-Malt1 complex, where TAK1 activates IKK leading to the above-mentioned IκBα phosphorylation^45^. Furthermore, activated Malt1 inactivates the negative regulator of NFκB signalling^7^.

Taken together, our experiments reported here provide substantial novel insight and mechanistic detail about various aspects of the inflammatory reaction, including (1) the *in vivo* role of neutrophil gene expression and chemokine/cytokine release; (2) the contribution of CARD9 to non-infectious inflammation and (3) the pathways coupling immunoreceptors to cellular effector functions (Supplementary Fig. 9). Those results will improve our understanding of the pathomechanism of autoimmune and inflammatory diseases and may contribute to the development of novel diagnostic and therapeutic strategies in the future.

## Methods

### Animals

Mice homozygous for the *Card9*^tm1Jrld^, *Bcl10*^tm1Mak^, *Malt1*^tm1Jrld^ or *Plcg2*^tm1JnI^ mutations (referred to as *Card9*^−/−^, *Bcl10*^−/−^, *Malt1*^−/−^ and *Plcg2*^−/−^ mutants, respectively) or triple homozygous for the *Hck*^tm1Hev^, *Fgr*^*tm1Hev*^ and *Lyn*^tm1Sor^ mutations (referred to as *Hck*^−/−^*Fgr*^−/−^*Lyn*^−/−^ or 3 × SFK KO mice) were described previously^8^,^46^,^47^,^48^,^49^. Heterozygous mice carrying a deleted Syk allele (*Syk*^tm1Tyb^, referred to as *Syk*^−^) were time-mated and their embryos were used to generate bone marrow chimeras with a *Syk*^−/−^ hematopoietic system as described^31^. Mice homozygous for the *Card9*^tm1a(EUCOMM)Hmgu^ knockout-first mutation^33^ (referred to as *Card9*^−/− (EUCOMM)^ mice) were obtained from the Wellcome Trust Sanger Institute (EUCOMM Project No. 44813). Mice carrying the *Card9*^tm1a(EUCOMM)Hmgu^ knockout-first mutation were also bred to the FLPeR deleter strain^50^ to remove the FRT-flanked knockout-first cassette generating the *Card9*^tm1c(EUCOMM)Hmgu^ floxed allele (referred to as *Card9*^flox^), followed by breeding to the MRP8-Cre transgenic mice^35^ to generate MRP8-Cre^+^*Card9*^flox/flox^ (referred to as *Card9*^ΔPMN^) mice with a neutrophil-specific CARD9 deletion. Mice carrying the KRN T-cell-receptor transgene^51^ were obtained from Diane Mathis and Christophe Benoist (Harvard Medical School) and maintained in heterozygous form by mating with C57BL/6 mice.

All transgenic mice were backcrossed to the C57BL/6 genetic background for at least six generations. Genotyping was performed by allele-specific PCR.

Wild-type control C57BL/6 mice were purchased from Charles River or the Hungarian National Institute of Oncology (Budapest, Hungary). NOD mice, as well as a congenic strain carrying the CD45.1 allele on the C57BL/6 genetic background (B6.SJL-*Ptprc*^a^) were purchased from the Jackson Laboratory.

Mice were kept in individually sterile ventilated cages (Tecniplast) in a conventional facility. All animal experiments were approved by the Animal Experimentation Review Board of the Semmelweis University.

Bone marrow chimeras were generated by intravenous injection of bone marrow cells into recipients carrying the CD45.1 allele on the C57BL/6 genetic background, which were lethally irradiated before by 11 Gy from a ^137^Cs source using a Gamma-Service Medical (Leipzig, Germany) D1 irradiator. Four weeks after transplantation, peripheral blood samples were stained for Ly6G and CD45.2 (at a final concentration of 2 μg ml^−1^) and analysed by flow cytometry as previously described^32^.

Mice of both genders at 2–6 months of age were used for the experiments.

### K/B × N serum-transfer arthritis

Mice carrying the KRN T-cell receptor transgene on the C57BL/6 genetic background were mated with NOD mice to obtain transgene-positive (arthritic) K/B × N and transgene-negative (non-arthritic) B × N mice^30^,^51^. The presence of the transgene was determined by allele-specific PCR and confirmed by phenotypic assessment. Blood was taken by retroorbital bleeding and sera from arthritic and control mice were pooled separately.

Arthritis was induced by a single intraperitoneal injection of 300 μl K/B × N (arthritic) or B × N (control) serum into intact mice or bone marrow chimeras, followed by daily assessment of arthritis severity for 2 weeks as described^30^. Visible clinical signs were scored on a 0–10 scale by two investigators blinded for the origin and treatment of the mice. Ankle thickness was measured by a spring-loaded caliper (Kroeplin).

To investigate serum-induced articular dysfunction, mice were placed on a custom-made (Charles River Hungary, Isaszeg, Hungary) wire grid, slowly flipped and the length of time the mice held on to the grid was recorded as described^30^. The obtained data were combined into ‘holding-on curves' for each mouse.

### Autoantibody-induced skin blistering model

The murine model of the human blistering skin disease epidermolysis bullosa acquisita was triggered by systemic administration of antibodies against collagen type VII(ref 25). The bacterial expression of GST fusion proteins containing the mCVIICr fragment of mouse collagen VII, its purification, the generation of polyclonal antiserum in rabbits and IgG fraction separation were carried out as previously described^25^,^52^. The reactivity of the antibody preparation was tested with His-tagged mCVIICr fragment of murine collagen VII using an enzyme-linked immunosorbent assay (ELISA) as described^52^. Normal rabbit IgG (Sigma) or PBS was used as control.

Twelve milligrams of pathogenic or control IgG per mouse was injected subcutaneously under isoflurane anaesthesia on days 0, 2, 4, 6 and 8 (60 mg total IgG/mouse). The disease onset and progression was followed by clinical assessment every second day. Scoring was based on the specific dermatological abnormalities (for example, erythema, blister, crust) and the size of the affected skin area, the latter one determined by using predefined percentages of the various areas of the skin of mice (for example, one ear: 2.5%; snout: 5% and so on). Antibody levels against collagen VII in blood samples at day 6 were determined by ELISA.

### *In vivo* analysis of myeloid cell migration

In case of the K/B × N serum-transfer model, the ankle or front paw area was flushed with 1 ml PBS supplemented with 10 mM EDTA (pH 7.5) and 20 mM HEPES (pH 7.4; Sigma) on day 4 to obtain a sample of synovial infiltrate. The number and distribution of the myeloid cells in the resulting suspension was then determined by flow cytometry. Cells were identified based on their forward- and side-scatter characteristics, as well as by positive staining of neutrophils for Ly6G (clone 1A8 from BD Biosciences; final concentration: 2 μg ml^−1^), or positive staining of monocytes/macrophages for F4/80 (Clone: A3-1, AbD Serotec; final concentration: 10 μg ml^−1^) or CD11b (Clone: M1/70, BD Biosciences; final concentration: 2 μg ml^−1^) within the Ly6G-negative gate.

A competitive migration assay in mixed bone marrow chimeras^30^ was also used to assess *in vivo* migration of myeloid cells. To this end, wild type (C57BL/6) or Card9^−/−^ bone marrow cells (all carrying the CD45.2 allele) were mixed at varying ratios with bone marrow cells from congenic mice expressing CD45.1 on the C57BL/6 genetic background. This mixed cell suspension was injected intravenously into lethally irradiated CD45.1-expressing recipient mice, giving rise to mixed bone marrow chimeras carrying CD45.2-expressing wild type or Card9^−/−^ along with CD45.1-expressing wild type hematopoietic cells within the same animals. After the repopulation of the bone marrow, the chimeras were subjected to K/B × N serum-transfer arthritis and their synovial infiltrates were prepared as described above. The percentage of CD45.2-expressing cells were analysed by flow cytometry as described above, with an additional staining for CD45.2 (Clone: 104, BD Biosciences; final concentration: 2 μg ml^−1^).

Relative migration (accumulation) of the CD45.2-positive neutrophils or monocytes/macrophages (relative to the CD45.1-expressing wild-type cells) was calculated as described^32^.

### Isolation and activation of neutrophils and macrophages

Mouse neutrophils were isolated from the bone marrow of the femurs and tibias of intact mice or chimeras by hypotonic lysis followed by Percoll (GE Healthcare) gradient centrifugation using sterile and endotoxin-free reagents as described^53^. Cells were kept at room temperature in Ca^2+^- and Mg^2+^-free medium until use and prewarmed to 37 °C prior to activation. Neutrophil assays were performed at 37 °C in Hanks' Balanced Salt Solution (HBSS) supplemented with 20 mM HEPES, pH 7.4. Neutrophil cell surface Fcγ receptor expression was detected by anti-FcγRII/III (Clone: 2.4G2, BD Biosciences; final concentration: 2 μg ml^−1^) and anti-FcγRIV antibodies (final concentration: 10.2 μg ml^−1^), followed by a secondary staining with fluorochrome-conjugated Mouse anti-rat and Mouse anti-hamster antibodies (Clones: MRK-1 and G70-204/G94-56, respectively, both from BD Biosciences; final concentration: 1 μg ml^−1^).

Mouse bone marrow-derived macrophages were obtained by culturing bone marrow cells from intact mice in α-MEM (Sigma) complemented with 10% FCS (Invitrogen), 2 mM L-glutamine, antibiotics, 10 mM HEPES (all from Sigma) and 10% conditioned medium from CMG 14-12 cells as a source of recombinant murine M-CSF^54^ for 8 days on bacterial Petri dishes with media changes every 2–3 days. Cells were suspended by 5 mM EDTA, washed and serum-starved for 2 h before the experiments.

To obtain immobilized immune complex-coated surfaces, human serum albumin (Sigma) or lactoferrin (Sigma) was either bound directly to regular or luminometric Nunc MaxiSorp F96 (Thermo Fisher) plates or covalently linked to poly-L-lysine-coated 24-well tissue culture plates or 6-cm tissue culture dishes and then treated with anti-human serum albumin or anti-lactoferrin IgG (Sigma) as described^55^.

For the analysis of cytokine release, 100 or 500 μl aliquots of 4 × 10^6^ per ml neutrophils or 500 μl aliquots of 3 × 10^6^ per ml macrophages suspended in HBSS supplemented with 20 mM HEPES, pH 7.4 were plated on immobilized immune complex surfaces in 96-well ELISA plates or 24-well tissue culture plates, stimulated for 6 h (neutrophils) or 24 h (macrophages) and the inflammatory mediator concentration of the supernatant was determined. For the analysis of LTB_4_ production, 100 μl aliquots of 4 × 10^6^ per ml neutrophils suspended in DMEM (Sigma) were plated on immobilized immune complex surfaces in 96-well ELISA plates for 1 h and the LTB_4_ concentration of the cell-free supernatant was measured. Superoxide release by neutrophils was followed by a cytochrome c reduction test from 100 μl aliquots of 4 × 10^6^ per ml cells plated on immune complex-coated surfaces in 96-well ELISA plates as described^56^. To test ROS production by macrophages, 100 μl aliquots of 3 × 10^6^ per ml cells were plated on immune complex-coated surfaces in 96-well luminometric ELISA plates in the presence of 50 μg ml^−1^ lucigenin (Sigma) and the chemiluminescence of the samples was followed by a Berthold (Bad Wildbad, Germany) Mithras LB 940 luminometer. The release of gelatinase was determined by gelatinase zymography as previously described^56^.

*In vitro* migration of neutrophils was tested by using a Transwell (Corning) assay with inserts of 3 μm pore size coated with human fibrinogen as described^30^. Three microlitres of fMLP (Sigma), 100 ng ml^−1^ MIP-2 (Peprotech) or 50 ng ml^−1^ LTB_4_ (Santa Cruz Biotechnology) was used as chemoattractants. Transmigrated cells were measured by an acid phosphatase assay^30^.

### Analysis of inflammatory mediators

The release of inflammatory mediators was tested from the cell-free supernatants of synovial infiltrates gathered on day 4 or of *in vitro* stimulated neutrophils or macrophages. The levels of IL-1β, KC, MIP-1α and MIP-2, as well as of the lipid mediator LTB_4_, were tested by commercial ELISA kits (R&D Systems) according to the manufacturer's instructions.

### Gene expression measurements

For *in vitro* gene expression studies, 500 μl aliquots of 4 × 10^6^ per ml neutrophils suspended in HBSS supplemented with 20 mM HEPES, pH 7.4 were plated on immobilized immune complex surfaces in 24-well tissue culture plates and stimulated for 4  h. The reaction was stopped on ice and the cells (adherent and nonadherent combined) were lysed and total RNA were prepared by the RNeasy Micro Kit (Qiagen). RNA samples were reverse transcripted to cDNA by the cDNA Synthesis Kit (Thermo Scientific) and were kept at −70 °C until use. The expression of unique genes was analysed using the TaqMan probes Mm01336189_m1 (*Il1b*), Mm00433859_m1 (*Cxcl1*), Mm00436450_m1 (*Cxcl2*), Mm00441258_m1 (*Ccl3*), Mm01182747 (*Alox5*), Mm00802100_m1 (*Alox5ap*), Mm00521826_m1 (*Lta4h*) and 4352932E (*Gapdh*) (all from Applied Biosystems) on a Roche LightCycler 480 device in a white 96-well plate. Global gene expression was measured by Affymetrix Microarrays at the UD GenoMed (Debrecen, Hungary) according to the manufacturer s instructions. More information and the raw data files are available in the Gene Expression Omnibus database under the accession number GSE78066.

### Biochemical studies

For analysis of Syk phosphorylation, 6 × 10^6^ neutrophils in 1.2 ml assay buffer were plated on immune complex-covered 6-cm tissue culture dishes and incubated for 10 min at 37 °C. The reaction was stopped on ice and the cells (adherent and nonadherent combined) were lysed in 100 mM NaCl, 30 mM Na-HEPES (pH 7.4), 20 mM NaF, 1 mM Na-EGTA, 1% Triton X-100, 1 mM benzamidine, freshly supplemented with 0.1 U ml^−1^ Aprotinin, 1:100 Mammalian Protease Inhibitor Cocktail, 1:100 Phosphatase Inhibitor Cocktail 2, 1 mM phenylmethylsulphonyl fluoride and 1 mM Na_3_VO_4_ (all from Sigma). After removal of insoluble material, lysates were either boiled in sample buffer or processed for immunoprecipitation. Immunoprecipitation of Syk was performed by an anti-Syk antibody (Clone: 5F5; BioLegend; final concentration: 4.4 μg ml^−1^) followed by a capturing step with Protein G-Agarose (Invitrogen) as described^57^,^58^,^59^.

For analysis of IκBα phosphorylation and degradation, 2 × 10^6^ neutrophils in 0.5 ml assay buffer were plated on immune complex-covered 24-well tissue culture plates and incubated for 20 min at 37 °C. The reaction was stopped on ice and the cells were lysed as described above. During the electrophoretic mobility shift assay, NFκB activation and nuclear translocation was followed using the same setup, but the cells were lysed with NP40 in the presence of dithiothreitol and phenylmethylsulphonyl fluoride. Nuclear extracts were prepared and were run on an acrylamide gel in the presence of infrared dye-labelled NFκB-binding-site-containing oligonucleotides (Licor) as described before^8^.

To test the efficacy of neutrophil-specific CARD9 deletion, neutrophils from mice of different genotypes were sorted on the basis of their Ly6G-staining on a FACSAria flow cytometer (BD Biosciences) from red blood cell-lysed bone marrow cell suspensions.

Whole cell lysates and immunoprecipitates were run on SDS–polyacrylamide gel electrophoresis and immunoblotted using antibodies against phosphotyrosine (Clone: 4G10, Merck Millipore; final concentration: 1 μg ml^−1^), Syk (Clone: N19, Santa Cruz Biotechnology; final concentration: 0.2 μg ml^−1^), IκBα (Cell Signaling; dilution: 1:1,000), phospho- IκBα (Cell Signaling, dilution: 1:1,000), CARD9 (Clone: H-90, Santa Cruz Biotechnology; final concentration: 0.2 μg ml^−1^), Bcl10 (Clone: H-197, Santa Cruz Biotechnology; final concentration: 0.2 μg ml^−1^) or β-actin (Clone: AC-74, Sigma; dilution: 1:5,000) followed by peroxidase-labelled secondary antibodies (GE Healthcare; dilution: 1:5,000). The signal was developed using the ECL system (GE Healthcare) and exposed to X-ray film. Western blots with more details are available in Supplementary Fig. 10.

### Presentation of the data and statistical analysis

Experiments were performed the indicated number of times. Quantitative graphs and kinetic curves show mean and s.e.m. from all independent *in vitro* experiments or from all individual mice from the indicated number of experiments. Unless otherwise stated, statistical analyses were carried out using two-way (factorial) ANOVA, with treatment and genotype being the two independent variables. In case of kinetic assays, area under the curve was used for statistical analysis. *P* values below 0.05 were considered statistically significant.

## Additional information

**How to cite this article:** Németh, T. *et al*. Neutrophil-specific deletion of the CARD9 gene expression regulator suppresses autoantibody-induced inflammation *in vivo*. *Nat. Commun.* 7:11004 doi: 10.1038/ncomms11004 (2016).

## Supplementary Material

Supplementary Information Supplementary Figures 1-10

Supplementary Table 1 Global gene expression changes during immune complex-induced neutrophil activation (wild type and Card9-/- cells).

## References

[b1] Mantovani A., Cassatella M. A., Costantini C. & Jaillon S. Neutrophils in the activation and regulation of innate and adaptive immunity. Nat. Rev. Immunol. 11, 519–531 (2011). 21785456 10.1038/nri3024

[b2] Németh T. & Mócsai A. The role of neutrophils in autoimmune diseases. Immunol. Lett. 143, 9–19 (2012). 22342996 10.1016/j.imlet.2012.01.013

[b3] Mócsai A. Diverse novel functions of neutrophils in immunity, inflammation, and beyond. J. Exp. Med. 210, 1283–1299 (2013). 23825232 10.1084/jem.20122220 PMC3698517

[b4] Yipp B. G. . Infection-induced NETosis is a dynamic process involving neutrophil multitasking *in vivo*. Nat. Med. 18, 1386–1393 (2012). 22922410 10.1038/nm.2847 PMC4529131

[b5] Scapini P. . The neutrophil as a cellular source of chemokines. Immunol. Rev. 177, 195–203 (2000). 11138776 10.1034/j.1600-065x.2000.17706.x

[b6] Scapini P. & Cassatella M. A. Social networking of human neutrophils within the immune system. Blood 124, 710–719 (2014). 24923297 10.1182/blood-2014-03-453217

[b7] Roth S. & Ruland J. Caspase recruitment domain-containing protein 9 signaling in innate immunity and inflammation. Trends Immunol. 34, 243–250 (2013). 23523010 10.1016/j.it.2013.02.006

[b8] Gross O. . Card9 controls a non-TLR signalling pathway for innate anti-fungal immunity. Nature 442, 651–656 (2006). 16862125 10.1038/nature04926

[b9] LeibundGut-Landmann S. . Syk- and CARD9-dependent coupling of innate immunity to the induction of T helper cells that produce interleukin 17. Nat. Immunol. 8, 630–638 (2007). 17450144 10.1038/ni1460

[b10] Robinson M. J. . Dectin-2 is a Syk-coupled pattern recognition receptor crucial for Th17 responses to fungal infection. J. Exp. Med. 206, 2037–2051 (2009). 19703985 10.1084/jem.20082818 PMC2737172

[b11] Glocker E. O. . A homozygous CARD9 mutation in a family with susceptibility to fungal infections. N Engl J Med 361, 1727–1735 (2009). 19864672 10.1056/NEJMoa0810719 PMC2793117

[b12] Drewniak A. . Invasive fungal infection and impaired neutrophil killing in human CARD9 deficiency. Blood 121, 2385–2392 (2013). 23335372 10.1182/blood-2012-08-450551

[b13] Roth S. . Rad50-CARD9 interactions link cytosolic DNA sensing to IL-1β production. Nat. Immunol. 15, 538–545 (2014). 24777530 10.1038/ni.2888 PMC4309842

[b14] Zhernakova A. . Genetic analysis of innate immunity in Crohn's disease and ulcerative colitis identifies two susceptibility loci harboring CARD9 and IL18RAP. Am. J. Hum. Genet. 82, 1202–1210 (2008). 18439550 10.1016/j.ajhg.2008.03.016 PMC2427314

[b15] McGovern D. P. . Genome-wide association identifies multiple ulcerative colitis susceptibility loci. Nat. Genet. 42, 332–337 (2010). 20228799 10.1038/ng.549 PMC3087600

[b16] Franke A. . Genome-wide meta-analysis increases to 71 the number of confirmed Crohn's disease susceptibility loci. Nat. Genet. 42, 1118–1125 (2010). 21102463 10.1038/ng.717 PMC3299551

[b17] Rivas M. A. . Deep resequencing of GWAS loci identifies independent rare variants associated with inflammatory bowel disease. Nat. Genet. 43, 1066–1073 (2011). 21983784 10.1038/ng.952 PMC3378381

[b18] Pointon J. J. . Elucidating the chromosome 9 association with AS; CARD9 is a candidate gene. Genes Immun. 11, 490–496 (2010). 20463747 10.1038/gene.2010.17 PMC2933507

[b19] Evans D. M. . Interaction between ERAP1 and HLA-B27 in ankylosing spondylitis implicates peptide handling in the mechanism for HLA-B27 in disease susceptibility. Nat. Genet. 43, 761–767 (2011). 21743469 10.1038/ng.873 PMC3640413

[b20] Arya R. . Genetic variants influencing joint damage in Mexican Americans and European Americans with rheumatoid arthritis. Genet. Epidemiol. 39, 678–688 (2015). 26498133 10.1002/gepi.21938

[b21] Kiryluk K. . Discovery of new risk loci for IgA nephropathy implicates genes involved in immunity against intestinal pathogens. Nat. Genet. 46, 1187–1196 (2014). 25305756 10.1038/ng.3118 PMC4213311

[b22] Hara H. . The adaptor protein CARD9 is essential for the activation of myeloid cells through ITAM-associated and Toll-like receptors. Nat. Immunol. 8, 619–629 (2007). 17486093 10.1038/ni1466

[b23] Heng T. S. . Immunological Genome Project, C. The Immunological Genome Project: networks of gene expression in immune cells. Nat. Immunol. 9, 1091–1094 (2008). 18800157 10.1038/ni1008-1091

[b24] Korganow A. S. . From systemic T cell self-reactivity to organ-specific autoimmune disease via immunoglobulins. Immunity 10, 451–461 (1999). 10229188 10.1016/s1074-7613(00)80045-x

[b25] Sitaru C. . Induction of dermal-epidermal separation in mice by passive transfer of antibodies specific to type VII collagen. J. Clin. Invest. 115, 870–878 (2005). 15841176 10.1172/JCI21386 PMC1070403

[b26] Liu Z. . A passive transfer model of the organ-specific autoimmune disease, bullous pemphigoid, using antibodies generated against the hemidesmosomal antigen, BP180. J. Clin. Invest. 92, 2480–2488 (1993). 7693763 10.1172/JCI116856 PMC288433

[b27] Chou R. C. . Lipid-cytokine-chemokine cascade drives neutrophil recruitment in a murine model of inflammatory arthritis. Immunity 33, 266–278 (2010). 20727790 10.1016/j.immuni.2010.07.018 PMC3155777

[b28] Wipke B. T. & Allen P. M. Essential role of neutrophils in the initiation and progression of a murine model of rheumatoid arthritis. J. Immunol. 167, 1601–1608 (2001). 11466382 10.4049/jimmunol.167.3.1601

[b29] Cremasco V., Graham D. B., Novack D. V., Swat W. & Faccio R. Vav/Phospholipase Cγ2-mediated control of a neutrophil-dependent murine model of rheumatoid arthritis. Arthritis Rheum. 58, 2712–2722 (2008). 18759305 10.1002/art.23757 PMC3004987

[b30] Jakus Z., Simon E., Frommhold D., Sperandio M. & Mócsai A. Critical role of phospholipase Cγ2 in integrin and Fc receptor-mediated neutrophil functions and the effector phase of autoimmune arthritis. J. Exp. Med. 206, 577–593 (2009). 19273622 10.1084/jem.20081859 PMC2699137

[b31] Jakus Z., Simon E., Balázs B. & Mócsai A. Genetic deficiency of Syk protects mice from autoantibody-induced arthritis. Arthritis Rheum. 62, 1899–1910 (2010). 20201079 10.1002/art.27438 PMC2972644

[b32] Kovács M. . The Src family kinases Hck, Fgr, and Lyn are critical for the generation of the *in vivo* inflammatory environment without a direct role in leukocyte recruitment. J. Exp. Med. 211, 1993–2011 (2014). 25225462 10.1084/jem.20132496 PMC4172222

[b33] Skarnes W. C. . A conditional knockout resource for the genome-wide study of mouse gene function. Nature 474, 337–342 (2011). 21677750 10.1038/nature10163 PMC3572410

[b34] Chiriac M. T. . NADPH oxidase is required for neutrophil-dependent autoantibody-induced tissue damage. J. Pathol. 212, 56–65 (2007). 17380558 10.1002/path.2157

[b35] Passegue E., Wagner E. F. & Weissman I. L. JunB deficiency leads to a myeloproliferative disorder arising from hematopoietic stem cells. Cell 119, 431–443 (2004). 15507213 10.1016/j.cell.2004.10.010

[b36] Abram C. L., Roberge G. L., Hu Y. & Lowell C. A. Comparative analysis of the efficiency and specificity of myeloid-Cre deleting strains using ROSA-EYFP reporter mice. J. Immunol. Methods 408, 89–100 (2014). 24857755 10.1016/j.jim.2014.05.009 PMC4105345

[b37] Elliott E. R. . Deletion of Syk in neutrophils prevents immune complex arthritis. J. Immunol. 187, 4319–4330 (2011). 21918195 10.4049/jimmunol.1100341 PMC3186826

[b38] Li L. . IL-17 produced by neutrophils regulates IFN-γ-mediated neutrophil migration in mouse kidney ischemia-reperfusion injury. J. Clin. Invest. 120, 331–342 (2010). 20038794 10.1172/JCI38702 PMC2798679

[b39] Monach P. A. . Neutrophils in a mouse model of autoantibody-mediated arthritis: critical producers of Fc receptor γ, the receptor for C5a, and lymphocyte function-associated antigen 1. Arthritis Rheum. 62, 753–764 (2010). 20191628 10.1002/art.27238 PMC3057458

[b40] Lai K. N. Pathogenesis of IgA nephropathy. Nat. Rev. Nephrol. 8, 275–283 (2012). 22430056 10.1038/nrneph.2012.58

[b41] Bakema J. E. & van Egmond M. The human immunoglobulin A Fc receptor FcαRI: a multifaceted regulator of mucosal immunity. Mucosal Immunol. 4, 612–624 (2011). 21937986 10.1038/mi.2011.36

[b42] Mócsai A., Walzog B. & Lowell C. A. Intracellular signalling during neutrophil recruitment. Cardiovasc. Res. 107, 373–385 (2015). 25998986 10.1093/cvr/cvv159 PMC4502828

[b43] Futosi K., Fodor S. & Mócsai A. Neutrophil cell surface receptors and their intracellular signal transduction pathways. Int. Immunopharmacol. 17, 638–650 (2013). 23994464 10.1016/j.intimp.2013.06.034 PMC3827506

[b44] Blonska M. & Lin X. NF-κB signaling pathways regulated by CARMA family of scaffold proteins. Cell Res. 21, 55–70 (2011). 21187856 10.1038/cr.2010.182 PMC3193407

[b45] Jiang C. & Lin X. Regulation of NF-κB by the CARD proteins. Immunol. Rev. 246, 141–153 (2012). 22435552 10.1111/j.1600-065X.2012.01110.x PMC3339759

[b46] Ruland J. . Bcl10 is a positive regulator of antigen receptor-induced activation of NF-κB and neural tube closure. Cell 104, 33–42 (2001). 11163238 10.1016/s0092-8674(01)00189-1

[b47] Wang D. . Phospholipase Cγ2 is essential in the functions of B cell and several Fc receptors. Immunity 13, 25–35 (2000). 10933392 10.1016/s1074-7613(00)00005-4

[b48] Lowell C. A., Soriano P. & Varmus H. E. Functional overlap in the *src* gene family: inactivation of *hck* and *fgr* impairs natural immunity. Genes Dev. 8, 387–398 (1994). 8125254 10.1101/gad.8.4.387

[b49] Chan V. W., Meng F., Soriano P., DeFranco A. L. & Lowell C. A. Characterization of the B lymphocyte populations in Lyn-deficient mice and the role of Lyn in signal initiation and down-regulation. Immunity 7, 69–81 (1997). 9252121 10.1016/s1074-7613(00)80511-7

[b50] Farley F. W., Soriano P., Steffen L. S. & Dymecki S. M. Widespread recombinase expression using FLPeR (flipper) mice. Genesis 28, 106–110 (2000). 11105051

[b51] Kouskoff V. . Organ-specific disease provoked by systemic autoimmunity. Cell 87, 811–822 (1996). 8945509 10.1016/s0092-8674(00)81989-3

[b52] Csorba K. . Cross-reactivity of autoantibodies from patients with epidermolysis bullosa acquisita with murine collagen VII. Cell. Mol. Life Sci. 67, 1343–1351 (2010). 20084423 10.1007/s00018-009-0256-3 PMC11115820

[b53] Mócsai A. . G-protein-coupled receptor signaling in Syk-deficient neutrophils and mast cells. Blood 101, 4155–4163 (2003). 12531806 10.1182/blood-2002-07-2346

[b54] Kertész Z. . Phospholipase Cγ2 is required for basal but not oestrogen deficiency-induced bone resorption. Eur. J. Clin. Invest. 42, 49–60 (2012). 21749368 10.1111/j.1365-2362.2011.02556.x PMC3266491

[b55] Jakus Z., Németh T., Verbeek J. S. & Mócsai A. Critical but overlapping role of FcγRIII and FcγRIV in activation of murine neutrophils by immobilized immune complexes. J. Immunol. 180, 618–629 (2008). 18097064 10.4049/jimmunol.180.1.618 PMC2647079

[b56] Németh T. . Neutrophil functions and autoimmune arthritis in the absence of p190RhoGAP: Generation and analysis of a novel null mutation in mice. J. Immunol. 185, 3064–3075 (2010). 20675588 10.4049/jimmunol.0904163 PMC3064944

[b57] Mócsai A. . Kinase pathways in chemoattractant-induced degranulation of neutrophils: The role of p38 mitogen-activated protein kinase activated by Src family kinases. J. Immunol. 164, 4321–4331 (2000). 10754332 10.4049/jimmunol.164.8.4321

[b58] Mócsai A. . The immunomodulatory adapter proteins DAP12 and Fc receptor γ-chain (FcRγ) regulate development of functional osteoclasts through the Syk tyrosine kinase. Proc. Natl Acad. Sci. USA 101, 6158–6163 (2004). 15073337 10.1073/pnas.0401602101 PMC395939

[b59] Mócsai A. . Integrin signaling in neutrophils and macrophages uses adaptors containing immunoreceptor tyrosine-based activation motifs. Nat. Immunol. 7, 1326–1333 (2006). 17086186 10.1038/ni1407 PMC4698344

