# Supplementary material for: Neutrophil-specific deletion of the CARD9 gene expression regulator suppresses autoantibody-induced inflammation in vivo
Source: Nat Commun. 2016 Apr 1;7:11004. doi: 10.1038/ncomms11004 (PMC4821996; doi:10.1038/ncomms11004)
Supplement: Supplementary Information — Supplementary Figures 1-10 [file ncomms11004-s1.pdf]

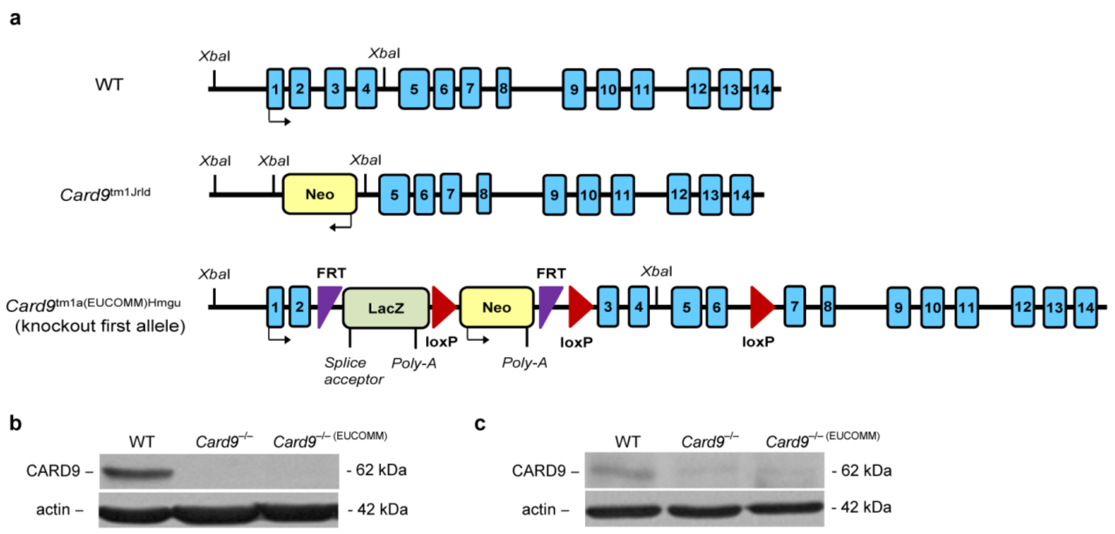

Supplementary Figure 1

Description and validation of the different Card9 deletion alleles

(a) The genomic organization of the wild type, *Card9*^{tm1Jrld} (referred to as *Card9*$^{-}$) and *Card9*$^{tm1a(EUCOMM)Hmgu}$ (referred to as *Card9*$^{-\ (EUCOMM)}$) alleles are shown. (b-c) CARD9 expression was determined by Western blot in the bone marrow (b) or the spleen (c) of wild type, *Card9*$^{-/-}$ or *Card9*$^{-/-\ (EUCOMM)}$ animals. The blots are representative of 2 independent experiments.

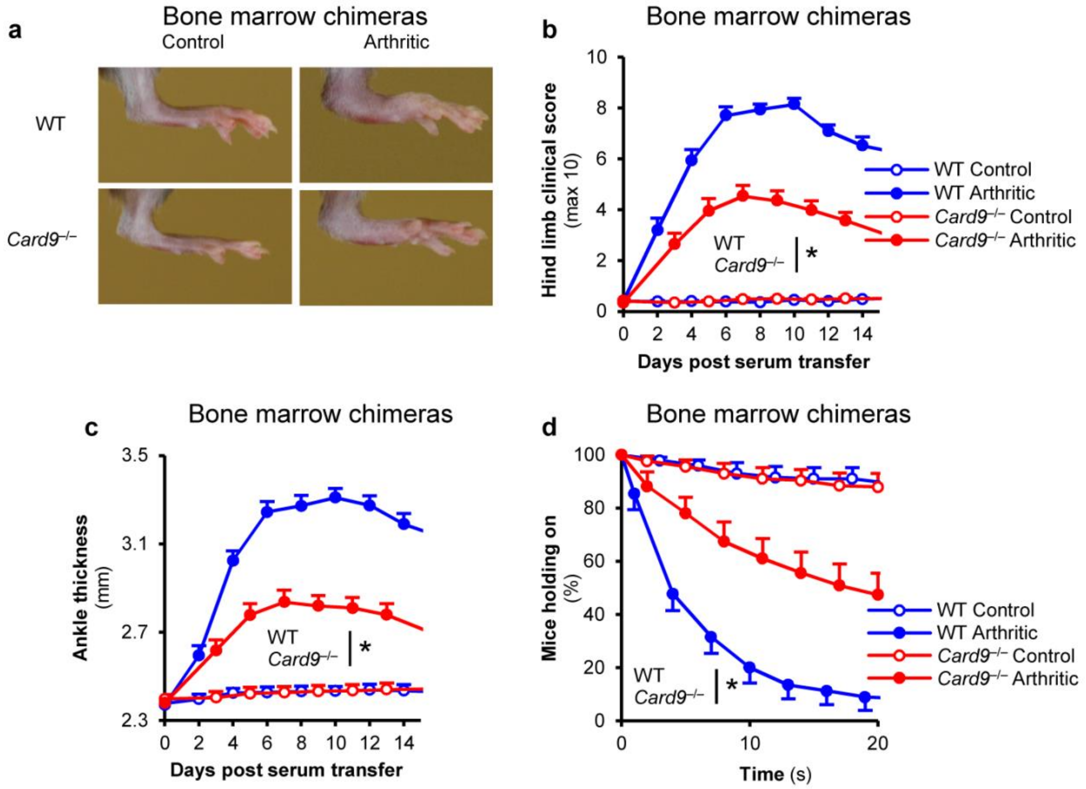

Supplementary Figure 2

Arthritis studies in Card9$^{-/-}$ bone marrow chimeras

Wild type (WT) and *Card9*$^{-/-}$ (a-d) bone marrow chimeras were injected with B×N (Control) or K/B×N (Arthritic) serum i. p. on Day 0. Arthritis development was followed by photographing on Day 7 (a), clinical scoring of the hind limbs (b), ankle thickness measurement (c) and an articular function test (hanging on a wire grid; (d)). Images are representative of, and quantitative data show mean and SEM from 17-18 control and 20-21 arthritic serum-treated individual mice per group from 7 independent experiments. Panel d results from functional test performed 12 times on each mouse between Days 7-10. * $p < 0.05$ (two-way ANOVA); see the text for actual p values.

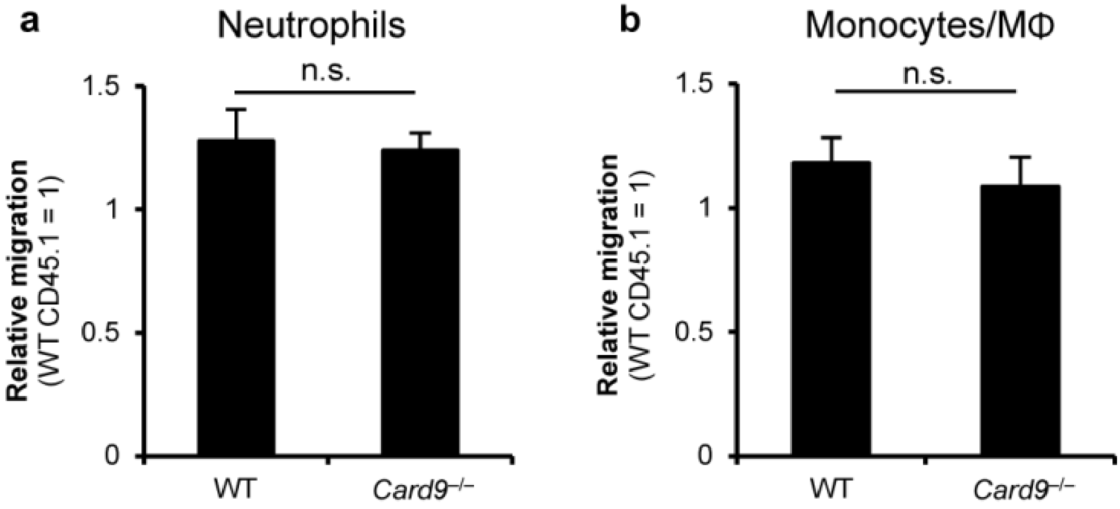

Supplementary Figure 3

Relative migration of wild type and Card9$^{-/-}$ neutrophils and monocytes/macrophages

Relative migration was calculated according to the formula mentioned in the Materials and methods section. Graphs show mean and SEM from 6-14 mice from 10 independent experiments (a) or 9 mice from 6 independent experiments (b). n.s., statistically not significant (Student's *t*-test); see the text for actual p values.

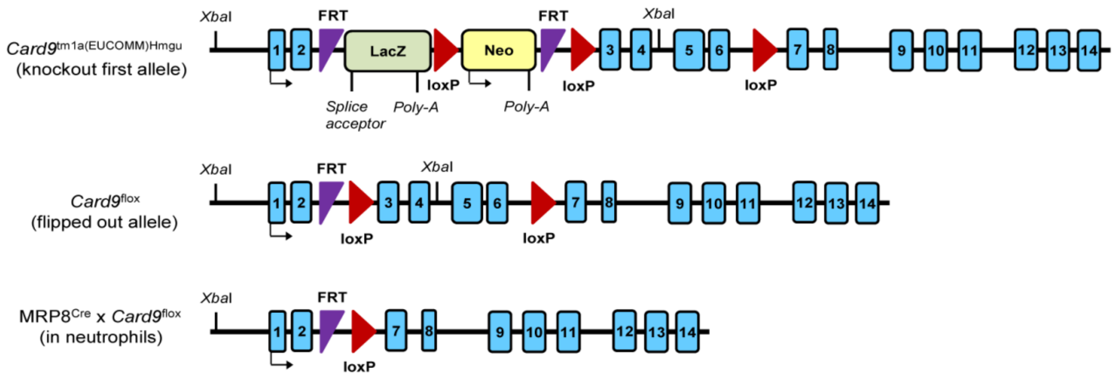

Supplementary Figure 4

Conditional deletion of Card9

This figure shows the genomic organization of the *Card9*$^{tm1a(EUCOMM)Hmgu}$ (referred to as *Card9*$^{-(EUCOMM)}$) allele, its flipped out version (referred to as *Card9*^{flox}) and the allele of the MRP8-Cre + *Card9*^{flox} combination in neutrophils.

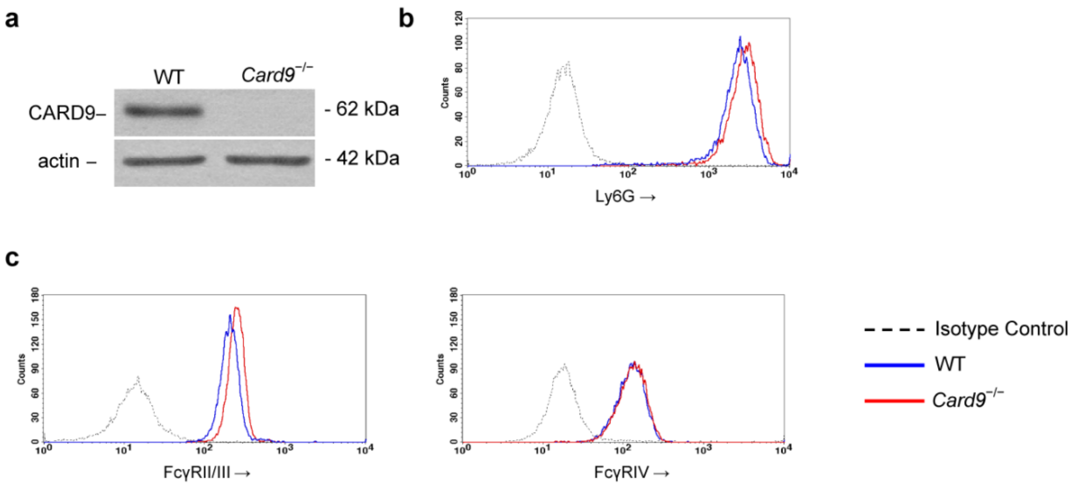

Supplementary Figure 5

Initial characterization of Card9$^{-/-}$ *neutrophils*

(a) Neutrophils specifically express CARD9 (shown by Western blot) and CARD9 deficiency does not alter neither their maturation (b), nor their activating Fcγ receptor cell surface expression (c). The blots and the flow cytometric histograms are representative of 3 independent experiments.

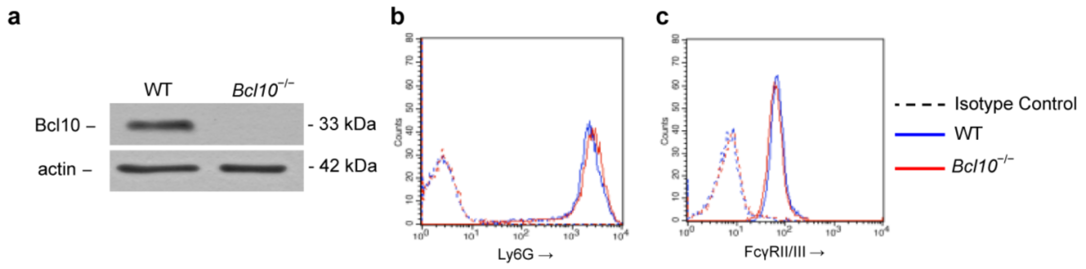

Supplementary Figure 6

Initial characterization of Bcl10$^{-/-}$ neutrophils

Neutrophils specifically express Bcl10 (a) and Bcl10-deficiency does not alter neither their maturation (b), nor their activating Fcγ receptor cell surface expression (c). The Western blot and the flow cytometric histograms are representative of 3 independent experiments.

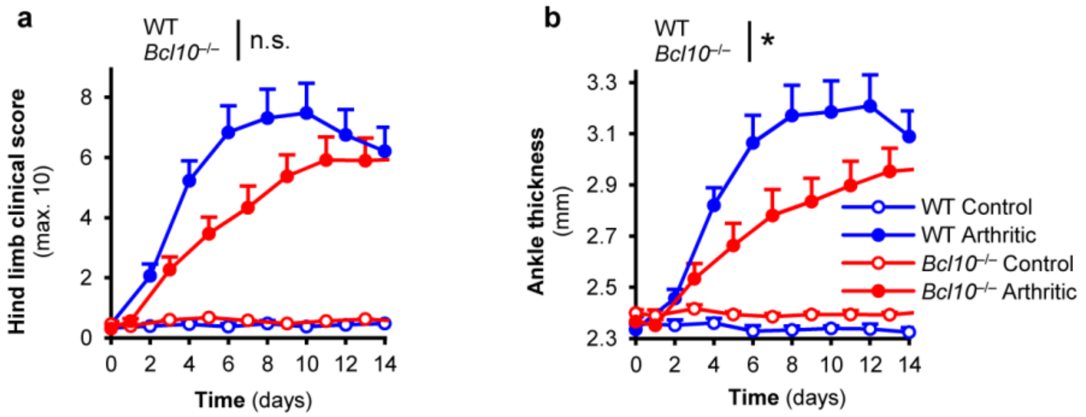

Supplementary Figure 7

Arthritis studies in Bcl10$^{-/-}$ *animals*

Wild type (WT) and *Bcl10*$^{-/-}$ (a-b) bone marrow chimeras were injected with B×N (Control) or K/B×N (Arthritic) serum i. p. on Day 0. Arthritis development was followed by clinical scoring of the hind limbs (a) and ankle thickness measurement (b). The quantitative data show mean and SEM from 12 mice per group from 2 independent experiments. * $p < 0.05$; n.s., statistically not significant (two-way ANOVA); see the text for actual p values.

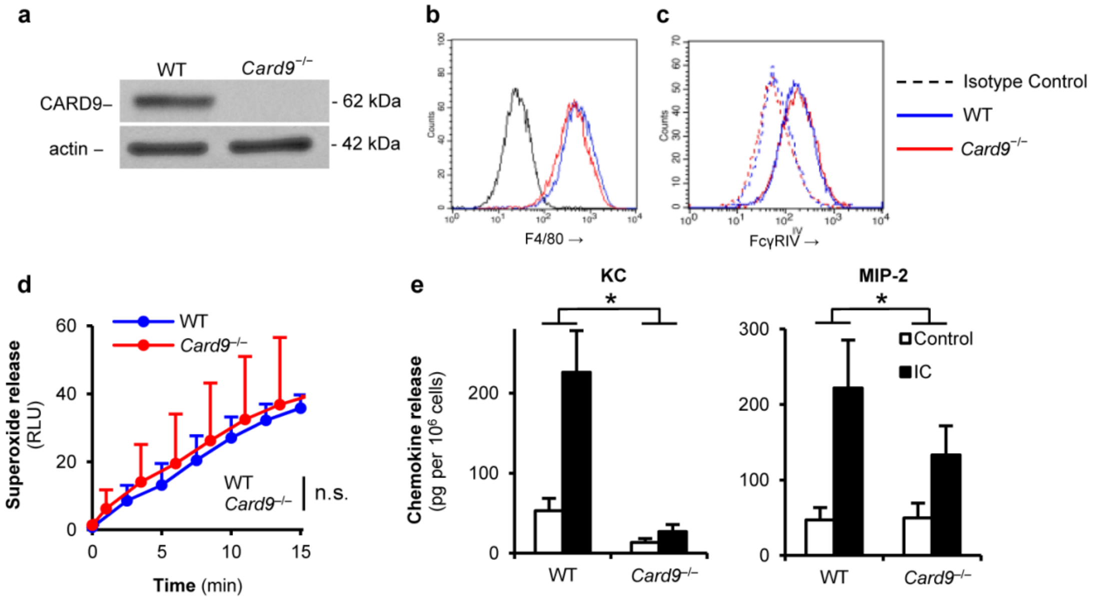

Supplementary Figure 8

Characterization of CARD9-deficient macrophages

Macrophages specifically express CARD9 (a) and CARD9-deficiency does not alter neither their maturation (b), nor their activating Fcγ receptor cell surface expression (c). The superoxide production of macrophages was measured by a luminometric method (d), while the proinflammatory mediator release was followed by ELISA assays from cell-free supernatants after a 24-hour incubation period (e). The Western blot and the flow cytometric histograms are representative of 3 independent experiments. Kinetic curves in Panel d and graphs in Panel e show mean and SEM of 3 independent experiments. In connection with kinetic curves, control data points were subtracted. * $p < 0.05$; n.s., statistically not significant (two-way ANOVA).

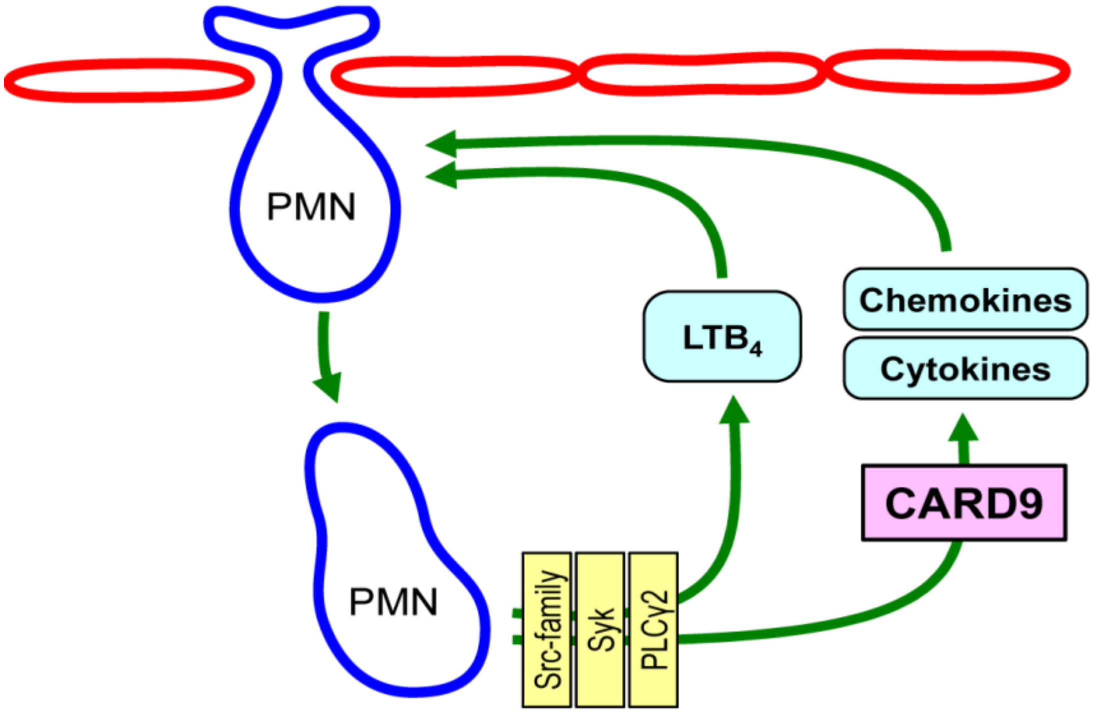

Supplementary Figure 9

Overview of the feedback regulation of neutrophil recruitment by CARD9

Neutrophils drive their own recruitment during sterile inflammation by releasing chemokines, cytokines and lipid mediators (LTB_4). Neutrophil CARD9 acts downstream of receptor-proximal Src-family kinases, Syk and PLCγ2 to trigger chemokine and cytokine but not LTB_4 release.

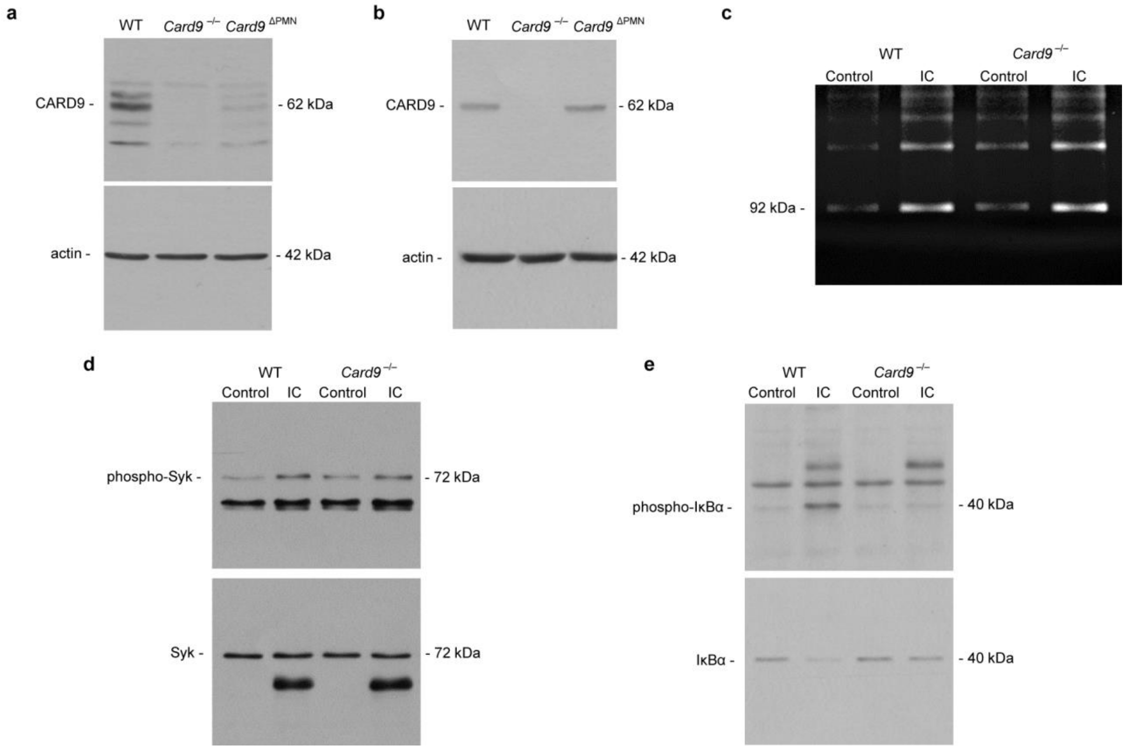

Supplementary Figure 10

Western blot images and the gelatinase zymogram with more details

(a-b) Western blot images showing CARD9-expression in neutrophil (a) or macrophage cell lysates (b) from Figs 5a-b. (c) Gelatinase zymogram of immune complex (IC)-activated CARD9-deficient mouse neutrophils from Fig. 6b. (d) Syk-phosphorylation in Card9$^{-/-}$ neutrophils upon activation through Fcγ receptors (see Fig. 7d). (e) The phosphorylation and degradation of IκBα in CARD9-deficient mouse neutrophils when plated on immune complexes (see Fig. 7i).
